# Evaluation of the physicochemical, metabolomic, and sensory characteristics of Chikso and Hanwoo beef during wet aging

**DOI:** 10.5713/ab.23.0001

**Published:** 2023-02-26

**Authors:** Dongheon Lee, Hye-Jin Kim, Azfar Ismail, Sung-Su Kim, Dong-Gyun Yim, Cheorun Jo

**Affiliations:** 1 Department of Agricultural Biotechnology, Center for Food and Bioconvergence, and Research Institute of Agriculture and Life Science, Seoul National University, Seoul 08826, Korea; 2 Institute of Green Bio Science and Technology, Seoul National University, Pyeongchang 25354, Korea; 3 Department of Animal Product Technology, Faculty of Animal Husbandry, Universitas Padjadjaran, West Java 45363, Indonesia

**Keywords:** Chikso, Hanwoo, Meat Quality, Metabolomics, Sensory Attribute, Wet Aging

## Abstract

**Objective:**

This study aimed to evaluate the physicochemical, metabolomic, and sensory qualities of Chikso and Hanwoo beef during 28 days of wet aging.

**Methods:**

Rump and loins from Hanwoo and Chikso were obtained and wet-aged for 28 days at 4°C. The samples were collected at 7-day interval (n = 3 for each period). Physicochemical qualities including pH, meat color, shear force value, and myofibrillar fragmentation index, metabolomic profiles, and sensory attributes (volatile organic compounds and relative taste intensities) were measured.

**Results:**

Chikso showed a significantly higher shear force value than Hanwoo on day 0; however, no differences between breeds were found after day 14, regardless of the cuts. Overall, Chikso had more abundant metabolites than Hanwoo, especially L-carnitine and tyrosine. Among the volatiles, the ketone ratio was higher in the Chikso rump than the Hanwoo rump; however, Chikso had fewer alcohols and aldehydes than Hanwoo. Chikso rump showed higher taste intensities than the Hanwoo rump on day 0, and sourness decreased in Chikso, but increased in the Hanwoo rump on day 14. Wet aging for 14 days intensified the taste of Chikso loin but reduced the umami intensity of Hanwoo loin.

**Conclusion:**

Chikso had different metabolomic and sensory characteristics compared to Hanwoo cattle, and 14 days of wet aging could improve its tenderness and flavor traits.

## INTRODUCTION

Korean native cattle are classified as four indigenous cattle breeds: Hanwoo, Chikso, Heugu, and Jeju black [1]. Among them, Hanwoo is a well-known breed to consumers owing to its superior meat quality, such as excellent tenderness, high degree of marbling, and desirable flavor [2]. Hanwoo has low connective tissue and high intramuscular fat content, leading to high marbling quality, that is important for determining meat quality grade in Korea and other countries [3,4]. As a result, the great consumer demand for Hanwoo beef is attributed to its tenderness and flavor.

On the other hand, the beef quality of other cattle breeds, especially Chikso, has not well been evaluated. Despite the limited scientific information on Chikso beef quality, the rarity of Chikso beef in the market due to the limited population of approximately 4,000 heads in restricted areas makes it captivating to consumers [5]. Recently, Chikso has gained attention owing to its unique flavor characteristics to consumers who prioritize desirable meat flavor and seek new meat products [2]. Previous studies have reported flavor differences between cooked Hanwoo and Chikso beef through sensory assessment or instrumental analyses [4,5]. Wei et al [6] emphasized that the provision of beef products with flavor differences from a variety of cattle breeds is important to attract consumers with different preferences. Therefore, it is necessary to investigate Chikso beef quality and its differentiated characteristics from common Hanwoo beef to diversify Korean native cattle products in response to a variety of consumer demands.

According to the consumers and suppliers of Chikso beef, the toughness of Chikso beef has been pointed out as one of its drawbacks. As wet aging of beef has been widely adopted in the meat industry to improve eating quality, such as meat tenderization and flavor development [7,8], applying wet aging to Chikso beef could be an effective strategy for value addition through improving tenderness and flavor to enhance organoleptic attributes. Various flavor compounds and metabolite profiles in meat can be changed during wet aging [9,10]. As flavor is constituted of volatile organic compounds (VOC) and taste-active compounds, the quantitative analysis of these flavor compounds and metabolites that act as flavor precursors can help interpret beef’s sensory quality [11–13]. In addition, the development of beef flavor can be assessed by instrumental sensory analysis, such as electronic tongue and nose, which has the benefit of objective analysis [14,15]. Therefore, a 28-day wet aging process was applied to Chikso and Hanwoo rump, and loin cuts to investigate the effect of wet aging on meat quality. Additionally, the physicochemical quality, metabolomic profiles, and sensory attributes of Chikso rump and loin cuts during wet aging were evaluated and compared with those of Hanwoo beef.

## MATERIALS AND METHODS

### Raw materials and wet aging process

Beef rump (M. semimembranosus) and loin (M. longissimus dorsi) were obtained from Hanwoo (32 months old, quality grade 1, 462 kg carcass weight) and Chikso (57 months old, quality grade 2, 373 kg carcass weight) at 48 h post-mortem, which were raised at the same condition (farm and diet). The age of each animal was determined based on its live weight in a commercial market. Visual fat and connective tissue were removed from the surface of the muscle, and each meat sample was cut into an average weight of 250 g.

To study the effect of wet aging on the Hanwoo and Chikso beef quality, rump and loin with an average weight of 250 g were vacuum-packaged (HFV-600L; Hankook Fujee Machinery Co., Ltd., Hwaseong, Korea) into low-density polyethylene/nylon bags (2 mL O^2^·(m^2^)^−1^·24 h^−1^ at 0°C, 0.09 mm thickness; Sunkyung Co., Ltd., Seoul, Korea). The wet aging process was continued at 4°C for 28 days, and samples were collected at 7-day intervals (n = 3 for each wet aging period). At each wet aging period (days 0, 7, 14, 21, and 28), the beef samples were examined for color, and the samples were ground and used for physicochemical quality analyses. The remaining samples were vacuum packaged and stored at −70°C until further analyses.

### Physicochemical quality analyses

#### pH

The pH of the meat sample was measured according to Lee et al [16]. One gram of each sample was homogenized with 9 mL of deionized distilled water using a homogenizer (T25 digital ULTRA-TURRAX; Ika Works, Staufen, Germany) at 9,600 rpm for 30 s. Then, the homogenates were centrifuged at 2,265×g for 10 min (Continent 512R; Hanil Co., Ltd., Daejeon, Korea). The supernatants were filtered (No. 4; Whatman International Ltd., Kent, UK) and the pH of each sample was measured using a pH meter. A pH meter (Seven2Go; Mettler Toledo Inc., Schwerzenbach, Switzerland) was pre-calibrated by standardized buffer solutions (pH 4.01, 7.0, and 9.21) at room temperature.

#### Meat color

Meat color was measured using a colorimeter (CM-5; Konica Minolta Censing Inc., Osaka, Japan) [16]. Before the analysis, the meat was bloomed for 30 min. Following pre-calibration of the colorimeter using a standard white plate, the analysis was conducted under the conditions of a D65 illuminant, a 10° standard observer, and a plate with a diameter of 30 mm. The meat’s lightness, redness, and yellowness were expressed as Commission Internationale d’Eclairage (CIE) L*, a*, and b* values, respectively. Six measurements were taken for each sample, and the average was used as one replicate.

#### Shear force value

The shear force value of Chikso and Hanwoo beef was measured according to Kim et al [17] with some modifications. Samples with an average weight of 100 g were placed in polyethylene bags in a water bath at 85°C until the internal temperature of the sample reached 72°C. After cooking, the samples were cooled to room temperature. Then, round cores were taken using a cork borer (1.27 cm diam.) parallel to the direction of the muscle fiber. A texture analyzer (TA1; AMETEK Lloyd Instruments Ltd., Fareham, UK) with a cross-head speed of 200 mm/min, a test speed of 60 mm/min, and a trigger load of 0.1 N was used.

#### Myofibrillar fragmentation index

The myofibrillar fragmentation index (MFI) analysis was conducted according to Kim et al [17] with a slight modification. The meat sample (1 g) was placed in the centrifugal tube and was homogenized at 15,000 rpm by adding 19 mL of MFI buffer (pH 7.0 at 4°C, containing 100 mM KCl, 1 mM ethylenediaminetetraacetic acid, 1 mM NaN_3_, and 25 mM potassium phosphate) for 30 s (T25 digital ULTRA-TURRAX; Ika Works, Germany). The homogenates were filtered through a 1-mm mesh strainer to remove connective tissues and washed with 10 mL of MFI buffer. The filtrate was centrifuged at 1,000×g for 15 min (Continent 512R; Hanil Co., Ltd., Korea). The supernatant was discarded, and the pellet was washed with 10 mL MFI buffer and vortexed. This experiment was repeated five times. Next, 10 mL of MFI buffer was added to the remaining pellet, vortexed, and the protein concentration was determined using the biuret method. The samples were then diluted with MFI buffer to reach 0.5 mg/mL of protein concentration. The absorbance of each sample was measured at 540 nm using a spectrophotometer (X-ma 3100; Human Co. Ltd., Seoul, Korea). The MFI value was calculated by multiplying the absorbance by 200.

#### Nuclear magnetic resonance-based metabolomic analysis

The nuclear magnetic resonance (NMR) analysis was conducted according to Kim et al [18]. The meat samples (5 g) were homogenized with 20 mL of 0.6 M perchloric acid at 16,000 rpm for 1 min (T25 digital ULTRA-TURRAX; Ika Works, Germany). The homogenates were centrifuged at 2,265×g for 20 min (Continent 512R; Hanil Co., Ltd., Korea). The pH of the collected supernatants was adjusted to 7.0 at room temperature using perchloric acid and potassium hydroxide solutions. The samples were centrifuged under the same conditions. Afterward, the samples were filtered (No. 1; Whatman International Ltd., UK), and the filtrates were lyophilized (Freezer dryer 18; Labco Corp., Kansas City, MO, USA). Following lyophilization, 1 mL of 20 mM deuterium oxide-based phosphate buffer (pH 7.0) containing 1 mM 3-(trimethylsilyl) propionic-2, 2, 3, 3-d_4_ acid (TSP) was added to the lyophilized samples. The reconstituted samples were incubated at 37°C for 10 min and centrifuged at 2,265×g for 20 min (Continent 512R; Hanil Co., Ltd., Korea). The supernatants were further centrifuged at 17,000×g for 10 min (HM-150IV; Hanil Co., Ltd., Korea). The supernatants were loaded into NMR tubes and used for further analysis.

One-dimensional ^1^H NMR and ^1^H-^13^C heteronuclear single quantum coherence (HSQC) spectra of the samples were recorded using a Bruker 850 MHz cryo-NMR spectrometer (Bruker Biospin GmbH, Rheinstetten, Germany). The spectra were manually corrected by Chenomx NMR suite 7.1 (Chenomx, Inc., Edmonton, AB, Canada). The peak was identified by comparing the peak spectrum of the sample from HSQC using Topspin 4.0.8 (Bruker Biospin GmbH, Germany) with that of the Human Metabolome Database (www.hmdb.ca). The identified peaks were quantified through Chenomx NMR Suite 7.1. The identification and quantification of each metabolite were referenced to the resonance of the TSP.

### Sensory attribute analyses

#### Volatile organic compound analysis

VOC analysis was conducted using solid-phase microextraction and gas chromatography-tandem mass spectrometry (SPME-GC-MS/MS), according to Lee et al [16] with a slight modification. Ground meat samples (5 g) were placed into a 20-mL headspace vial and sealed with a PTFE-faced silicone septum. The samples were incubated at 40°C for 10 min before the extraction. A polydimethylsiloxane/divinylbenzene fiber (Supelco Inc., Bellefonte, PA, USA) was injected into the headspace of the vial and the extraction process was continued at 40°C for 30 min. The extracted VOCs were desorbed using a GC injector (Trace 1310; Thermo Fisher Scientific, Waltham, MA, USA) for 2 min at a split ratio of 1:10. A DB-Wax column (60 m×0.25 mm i.d., and 0.50 μm film thickness; Agilent Technologies Inc., Santa Clara, CA, USA) was equipped to the GC for VOCs separation. The GC condition was as follows: helium was used as the carrier gas at the flow rate of 1.5 mL/min; initial oven temperature was held at 40°C for 2 min, increased at a rate of 4°C/min and held at 150°C for 10 min, then increased at a rate of 4°C/min and held at 200°C for 5 min, and finally, increased at a rate of 10°C/min and held at 230°C for 5 min. Mass spectra of the fragmentations were obtained using a triple quadrupole mass spectrometer (TSQ 8000; Thermo Fisher Scientific, USA) in the electron impact (EI) mode with a scan range from 35 to 550 m/z in full-scan mode at 0.2 s of scan interval. VOCs in Chikso and Hanwoo beef were identified by comparing their mass spectra with those from the National Institute of Standards and Technology (NIST) mass spectral library (version 2.0 g) and the linear retention index (LRI). For the LRI, a *n*-alkane standard (C_8_–C_20_) was analyzed under the same conditions for the calculation of the LRI of each VOC.

#### Electronic tongue analysis

Electronic tongue analysis was performed according to Lee et al [14] with slight modifications. The ground meat samples (5 g) were homogenized with the addition of 100 mL deionized distilled water at 10,000 rpm for 30 s (T25 digital ULTRA-TURRAX; Ika Works, Germany). The samples were centrifuged at 2,265×g for 10 min (Continent 512R; Hanil Co., Ltd., Korea) and the supernatants were filtered (No. 4; Whatman International Ltd., UK). Taste attributes of the samples were analyzed using an electronic tongue (Astree; Alpha MOS, Toulous, France). Taste screening was performed using Alpha soft (Alpha MOS, France) to compare the relative taste intensities of the samples using the following equation:


$$\text{Relative taste intensity}=(2.5\pm \frac{X-m}{\sigma })\times 2$$

where *X* and *m* are the mean sensor values for each and the total group, respectively, and *σ* represents the deviation of the sensor value within the total group. Relative taste intensity was measured on a 10-point scale. For the AHS and NMS sensors, the value $\frac{X-m}{\sigma }$ was subtracted from 2.5, as lower sensor values of AHS and NMS indicate higher taste intensities.

### Statistical analysis

All experiments were conducted in triplicate, and statistical analyses were performed via one-way analysis of variance using a linear model (SAS 9.4, SAS Institute Inc., Cary, NC, USA). Significant differences between treatments were determined using Tukey’s multiple comparison test (p<0.05). The results are expressed as the mean values and standard error of the mean. Correlation analysis was performed with SAS 9.4 (SAS Institute, Inc., USA). The heat map was visualized using TBtools v0.6735 (www.github.com/CJ-Chen/TBtools). For multivariate analysis, orthogonal partial least squares-discriminant analysis (OPLS-DA) and partial least squares-discriminant analysis (PLS-DA) were performed using MetaboAnalyst 5.0 (www.metaboanalyst.ca). OPLS-DA was performed between Hanwoo and Chikso beef for the entire wet aging period (days 0 to 28) to identify universally differentiated metabolites and VOC profiles between the two breeds. PLS-DA between Hanwoo and Chikso beef at each wet aging period was performed to study the effects of breeds and wet aging on metabolites and VOC changes.

## RESULTS

### Physicochemical characteristics of Hanwoo and Chikso beef during wet aging

#### pH

**Figure 1:**
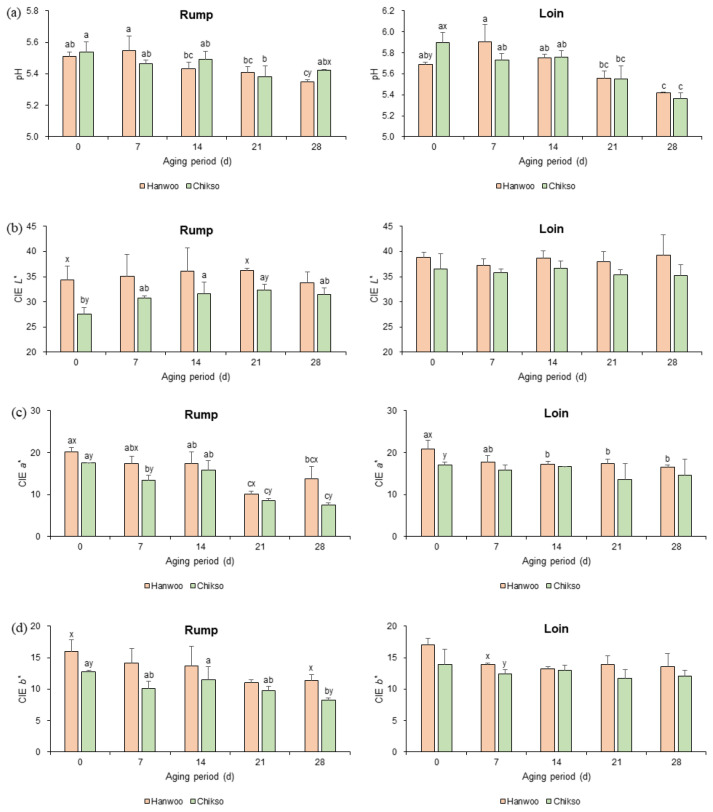
The pH (a), CIE L* (b), CIE a* (c), and CIE b* (d) values of Hanwoo and Chikso beef cuts during the wet aging period. CIE, Commission Internationale d’Eclairage. ^a–c^ Different letters within the same breed indicate significant differences (p<0.05). ^x,y^ Different letters within the same wet aging period indicate significant differences (p<0.05).

The pH of Hanwoo and Chikso rumps on day 0 was not significantly different, and they gradually decreased until day 21 (Figure 1a). However, on day 28, the Chikso rump showed a significantly higher pH than the Hanwoo rump. Generally, the meat pH drops during wet aging because of the accumulation of lactic acid by the increase in lactic acid bacteria under anaerobic conditions [11]. On the contrary, the generation of alkaline products such as ammonia and amines by protein hydrolysis during wet aging would increase the pH [19]. Meanwhile, Chikso loin had a significantly higher pH compared to Hanwoo loin on day 0. However, no significant difference in loin pH was observed between the two breeds after wet aging.

#### Meat color

During wet aging, the L* value of the Chikso rump significantly increased on days 14 and 21 compared to day 0 (Figure 1b). The increase in L* value in wet-aged beef may be attributed to the expulsion of moisture at the meat surface, which reflects the light, modification of surface protein structure by hydrolysis, denaturation during wet aging, etc. [7,20]. On the other hand, the Hanwoo rump had not exhibited any difference in L* value during 28 days of wet aging.

During wet aging, a* values of rump from both breeds significantly decreased (Figure 1c), following the results of the study performed by Ma et al [21]. This was possibly due to the reduced color stability by the oxidation of myoglobin and lipids [20]. It was also reported that extended vacuum storage would deteriorate color stability and bloom development of meat [21]. Meanwhile, a major difference in meat color between Hanwoo and Chikso rump was found in a* value. The Hanwoo rump showed a redder surface than the Chikso rump during wet aging (p<0.05), except on day 14. The a* value is highly affected by the content and chemical status of myoglobin [22]. Furthermore, recent studies found a negative correlation between color stability and phenylalanine or tryptophan, which act as substrates for reactive oxygen species, suggesting that metabolomic changes might influence the meat color [12,22]. In the present study, Chikso rump had higher phenylalanine content during wet aging compared to Hanwoo rump (Table 1), and phenylalanine was negatively correlated with a* value (*r* = −0.80; Supplementary Table S3). Therefore, it can be assumed that the high phenylalanine content in Chikso rump could decrease the a* value during storage.

The b* value in the Chikso rump decreased during wet aging (p<0.05), whereas there was no significant difference in Hanwoo rump (Figure 1d). However, the Hanwoo rump had a higher b* value on days 0 and 28 than the Chikso rump. In contrast, there were fewer changes in the color of Hanwoo and Chikso loins during wet aging.

#### Meat tenderness

**Figure 2:**
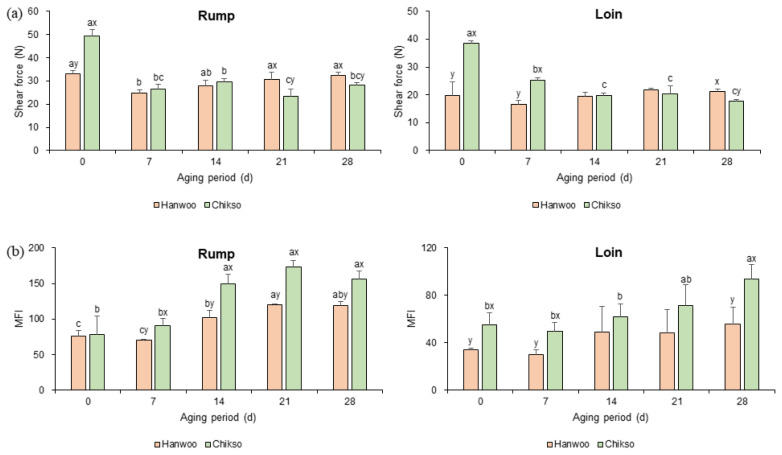
The shear force values (a) and MFI (b) of Hanwoo and Chikso beef cuts during the wet aging period. MFI, myofibrillar fragmentation index. ^a–c^ Different letters within the same breed indicate significant differences (p<0.05). ^x,y^ Different letters within the same wet aging period indicate significant differences (p<0.05).

Before wet aging, Chikso beef showed a significantly higher shear force value than Hanwoo rump (Figure 2a). Similarly, the shear force value of Chikso loin was higher than that of Hanwoo loin on day 0 (p<0.05). Hanwoo cattle have been bred for a long time to enhance meat tenderness, intramuscular fat content, marbling score, etc. [23]. As a result, Hanwoo beef has been recognized as tender beef with high meat quality grade, thin muscle fibers, and connective tissues [5]. However, the muscle characteristics of Chikso beef have not been well studied. It has been reported that the differentially differentiated genes between Hanwoo and Chikso were associated with adipose tissue development, fatty acid metabolism, and muscle lipid composition [24]. Therefore, differences in the characteristics of muscle tissue of Hanwoo and Chikso beef might be a possible candidate for explaining their different tenderness.

Nonetheless, as wet aging proceeded, the shear force value of the Chikso rump decreased to almost half of the original value on day 21. No significant difference was found between the Hanwoo and Chikso rumps after day 7. The Chikso rump showed lower shear force values on days 21 and 28 than the Hanwoo rump (p<0.05). The decrease in shear force value during wet aging may be attributed to the action of proteolytic enzymes [10]. Moreover, the effect of wet aging on meat tenderization was more remarkable in Chikso rump than in Hanwoo rump. Based on the standard of Belew et al [25] Chikso and Hanwoo rump on day 0 could be classified as “tough” (>45 N) and “tender” (between 31 and 38 N), respectively. However, after wet aging process, the shear force values of rump cut from two breeds showed similar values with the same category of “very tender” (<31 N), suggesting that Chikso rump might have similar tenderness properties with Hanwoo rump after day 7 of wet aging.

Similarly, the shear force values of the Chikso loin was significantly higher than the Hawnoo loin in the early phase of wet aging (days 0 to 7). However, as the wet aging process extended to day 14, the shear force value of the Chikso loin reached a value similar to that of the Hanwoo loin (p>0.05). Then, it significantly decreased and became lower than that of the Hanwoo loin on day 28. However, unlike the Chikso loin, there was no significant change in the shear force value of the Hanwoo loin during the wet aging period. Initially, Chikso loin could be categorized as “intermediate” (between 38 and 45 N) on day 0 [25]. Nonetheless, its shear force value reduced below 31 N after day 7 and showed insignificant values with Hanwoo loin on day 14, indicating that 14 days of wet aging might lead Chikso loin to show similar tenderness with Hanwoo loin.

Meat tenderness is mainly determined by the connective tissue content, myofibrillar protein degradation after slaughter, and sarcomere length [23,26]. Among them, the degree of myofibril degradation can be assessed by MFI. Especially, MFI value is more direct than shear force value for estimating the tenderization effect of wet aging on meat product [27]. In the present study, the MFI values of Hanwoo and Chikso rumps significantly increased with the wet aging period (Figure 2b), indicating that wet aging had a positive effect on the tenderization of Hanwoo and Chikso beef. Especially, the MFI value of Chikso rump was significantly higher than that of the Hanwoo rump after day 7. In the case of loin cuts, the MFI value of Chikso loin significantly increased, whereas that of Hanwoo loin did not change during wet aging. The results showed that Chikso beef with low tenderness on day 0 was significantly improved by wet aging. Furthermore, aging was more effective for Chikso beef than for Hanwoo beef. Therefore, wet aging for 14 days or more may contribute to the value addition of Chikso beef by tenderizing it to make the tenderness similar to that of Hanwoo beef.

### Comparison of metabolomic properties between two breeds during wet aging

A total of 27 metabolites were identified in rump and loin from both breeds during 28 days of wet aging, including 17 free amino acids, dipeptides and their derivatives, four nucleotides and nucleosides, four organic acids, and others (ethanol and niacinamide; Tables 1–4).

On day 0, Chikso rump had a higher abundance of tyrosine, L-carnitine, o-acetylcarnitine, fumarate, and ethanol than Hanwoo rump (Tables 1 and 2; p<0.05). As wet aging proceeded, the amounts of free amino acids, dipeptides, and derivatives increased significantly in both Hanwoo and Chikso rumps owing to the myofibrillar protein degradation (Table 1). In particular, higher amounts of alanine, asparagine, glutamate, glycine, isoleucine, leucine, methionine, phenylalanine, tyrosine, valine, and L-carnitine were found in the Chikso rump than in the Hanwoo rump (p<0.05). A higher MFI in the Chikso rump during wet aging might lead to higher amounts of free amino acids, dipeptides, and derivatives compared to the Hanwoo rump (Figure 2b). Among them, free amino acids can be utilized as flavor precursors for both VOC production and taste-active compounds [14,28]. Hence, metabolomic differences lead to the flavor differentiation between two breeds.

**Figure 3:**
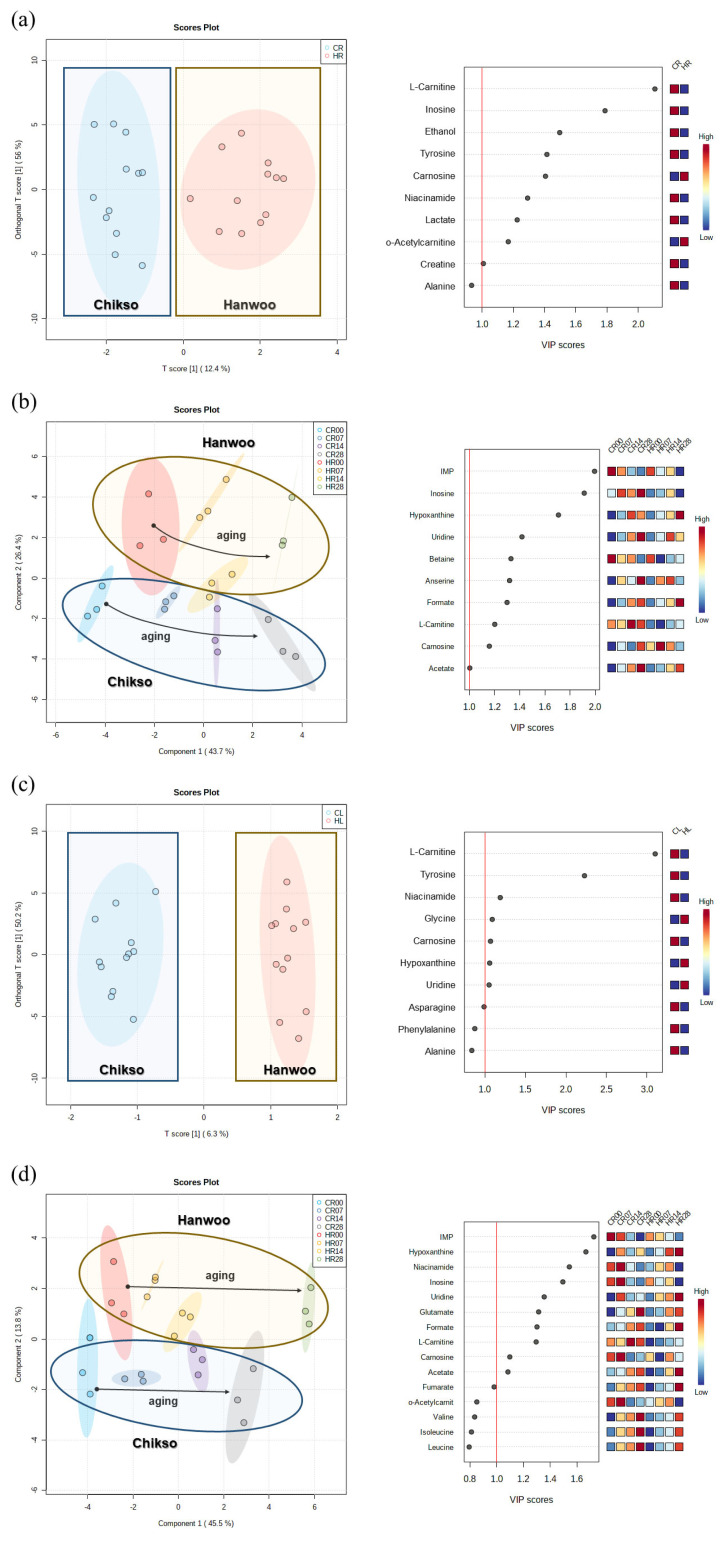
Multivariate analyses and variable importance in projection (VIP) for each analysis for the discrimination of metabolite profile between Hanwoo and Chikso beef during wet aging. Orthogonal partial least squares-discriminant analysis (OPLS-DA) between Hanwoo and Chikso beef for whole wet aging period (days 0 to 28) (a) and partial least squares-discriminant analysis (PLS-DA) between Hanwoo and Chikso rump at each wet aging period (b). Multivariate analyses were performed on beef loins in the same way as beef rumps (c-d). Black arrows in the PLS-DA plot illustrate the direction of metabolomic change of Hanwoo and Chikso beef during the wet aging period. The red-blue color system was used to represent the relative abundance of each metabolite in the beef sample. The numbers written in each class indicate the wet aging period of beef samples. CL, Chikso loin; CR, Chikso rump; HL, Hanwoo loin; HR, Hanwoo rump. Multivariate analyses and variable importance in projection (VIP) for each analysis for the discrimination of metabolite profile between Hanwoo and Chikso beef during wet aging. Orthogonal partial least squares-discriminant analysis (OPLS-DA) between Hanwoo and Chikso beef for whole wet aging period (days 0 to 28) (a) and partial least squares-discriminant analysis (PLS-DA) between Hanwoo and Chikso rump at each wet aging period (b). Multivariate analyses were performed on beef loins in the same way as beef rumps (c–d). Black arrows in the PLS-DA plot illustrate the direction of metabolomic change of Hanwoo and Chikso beef during the wet aging period. The red-blue color system was used to represent the relative abundance of each metabolite in the beef sample. The numbers written in each class indicate the wet aging period of beef samples. CL, Chikso loin; CR, Chikso rump; HL, Hanwoo loin; HR, Hanwoo rump.

The metabolomic profiles of the Hanwoo and Chikso rumps measured during the entire wet aging period were separated in the OPLS-DA plot, and major metabolomic differences between the two breeds were found to be L-carnitine, inosine, ethanol, tyrosine, carnosine, niacinamide, lactate, o-acetylcarnitine, and creatine, which had variable importance in projection (VIP) score higher than 1 (Figure 3a). L-carnitine is a bioactive compound that is responsible for fatty acid metabolism [29]. o-Acetylcarnitine in beef rump showed negative correlations with sour and bitter taste intensities measured via electronic tongue (Supplementary Table S4). Tyrosine is associated with a bitter taste; however, it could also enhance the umami intensity when salts and free acidic amino acids are present [8,30]. Meanwhile, the effects of both breed and wet aging period on the change in metabolomic profiles in beef rump were also explored through PLS-DA (Figure 3b). In the PLS-DA plot, both Hanwoo and Chikso rumps moved in a positive direction on the X axis during wet aging. The separation of the two breeds was attributed to the presence of free amino acids, L-carnitine, o-acetylcarnitine, and inosine. inosine 5′-monophosphate (IMP) and inosine, which had VIP>1 and thus had a great influence on the differentiation of sample groups in the PLS-DA plot, were also more abundant in the Chikso rump than in the Hanwoo rump (p<0.05). IMP can also contribute to the umami flavor of meat in addition to glutamate [13]. Therefore, higher amounts of glutamate and IMP in Chikso rump may act as umami flavor enhancers. In particular, the IMP content was negatively correlated with the intensity of the NMS sensor in the electronic tongue, suggesting the action of IMP in improving the umami taste of beef rump (*r* = −0.54; Supplementary Table S4). Overall, various metabolites were significantly increased in the Chikso rump during the wet aging process compared to the Hanwoo rump, which indicates that the sensory attributes of the Chikso rump may be enhanced by the increase in flavor precursors such as L-carnitine, o-acetylcarnitine, tyrosine, IMP, and inosine, and therefore be more distinguishable from the Hanwoo rump after wet aging.

In the loin cut, only the fumarate and ethanol contents were significantly higher in Chikso than in Hanwoo on day 0 (Table 4). However, on day 7, most metabolites, such as alanine, asparagine, isoleucine, leucine, phenylalanine, tyrosine, valine, anserine, carnosine, creatine, L-carnitine, o-acetylcarnitine, hypoxanthine, IMP, and inosine, were higher in Chikso loin than in Hanwoo loin (Tables 3 and 4; p<0.05). Remarkably, tyrosine and L-carnitine contents were significantly higher in the Chikso loin than in the Hanwoo loin during most of the wet aging period.

In the OPLS-DA plot, the major components that contributed to the separation between the two breeds included L-carnitine, tyrosine, niacinamide, glycine, carnosine, hypoxanthine, and uridine, which had a VIP>1 (Figure 3c). In particular, L-carnitine and tyrosine showed relatively high values of VIP scores, not only in the Chikso loin but also in the Chikso rump, indicating that these metabolites might be representative compounds in the Chikso beef. In the PLS-DA plot, metabolites in both Hanwoo and Chikso loins generally increased during the wet aging period (Figure 3d). As a result, both Hanwoo and Chikso loin moved in a positive direction along the X axis during the wet aging period in the PLS-DA plot. Similar to the results for beef rump, nucleosides and nucleotides, such as IMP, hypoxanthine, and inosine, were the major contributors to the separation of groups (VIP>1) in the PLS-DA plot. From the results, the differences in the metabolomic profiles between Hanwoo and Chikso beef, such as L-carnitine and tyrosine were observed. These differences may lead to the differentiation of sensory attributes between Hanwoo and Chikso beef.

### Sensory attributes between Hanwoo and Chikso beef during wet aging

#### Volatile organic compound analysis

**Figure 4:**
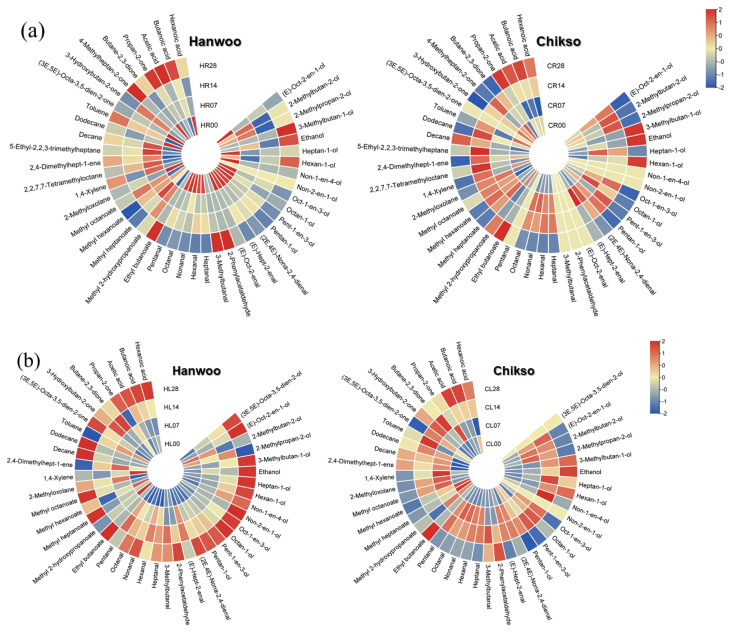
Heatmap of the volatile compound profile of Hanwoo and Chikso rump (a) and loin (b) during wet aging. The red-blue color system was used to represent the relative abundance of each volatile organic compound in the beef sample. The numbers written in each class indicate beef samples’ wet aging period (d) of beef samples. CL, Chikso loin; CR, Chikso rump; HL, Hanwoo loin; HR, Hanwoo rump.

**Figure 5:**
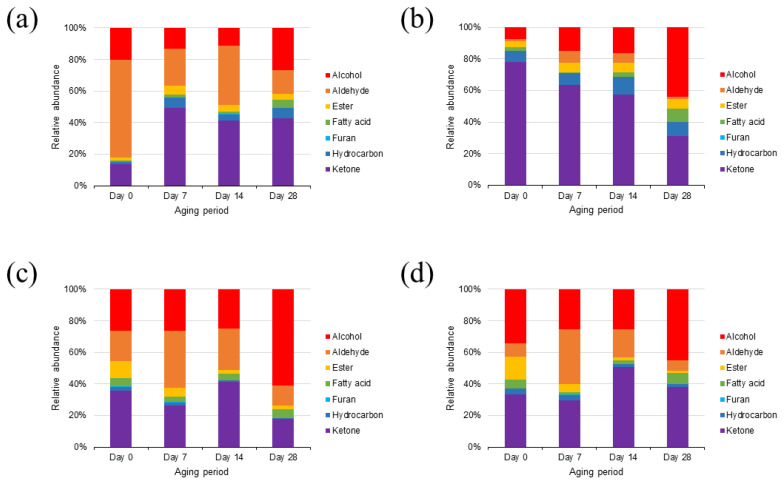
Relative abundances (%) of volatile organic compounds in the rump of Hanwoo (a) and Chikso (b) and the loin of Hanwoo (c) and Chikso (d) during wet aging, respectively. The compounds were assigned to alcohol, aldehyde, ester, fatty acid, furan, hydrocarbon, and ketone according to their functional groups.

A total of 43 VOCs were identified in the rump cuts during wet aging, including 12 alcohols, 10 aldehydes, 5 esters, 1 furan, 7 hydrocarbons, 5 ketones, and 3 volatile fatty acids (Figure 4). Most of the VOCs detected in this study have been reported as compounds produced by lipid oxidation [3]. Interestingly, on day 0, the major VOC group in the Hanwoo rump was aldehydes, whereas that in the Chikso rump was ketones (Figure 5a, b). In general, aldehydes are known as the major contributors to beef flavor among lipid oxidation-derived VOCs [7]. However, ketones also exhibit distinct oily, fruity, and fatty flavors with low odor-thresholds [11,31]. Therefore, these compounds could contribute significantly to the volatile flavor of Chikso rump. Ketones are known to be generated by amino acid degradation, lipid oxidation, carbohydrate metabolism or β-keto acid oxidation [32]. In particular, the amount of 3-hydroxybutan-2-one was significantly higher in the Chikso rump than in the Hanwoo rump, and it was the most abundant compound in the Chikso rump on day 0 (Supplementary Table S1). Therefore, 3-hydroxybutan-2-one is considered a potential contributor to the unique flavor of Chikso rump. Furthermore, 4-methylheptan-2-one was present only in the Chikso rump until day 21. In contrast, other VOC groups, such as alcohols, aldehydes, and esters, were present at higher concentrations in the Hanwoo rump than in the Chikso rump. Hexanal was the most abundant compound in the Hanwoo rump. It is a product of linoleic acid oxidation and is a major VOC in beef that gives a pleasant flavor to cooked meat [7,8]. Moreover, the degradation products of oleic acid, such as heptanal, octanal, and nonanal, were also detected in the Hanwoo and Chikso rumps. These compounds have sweet and fatty odor characteristics and influences the beef flavor significantly [11,33]. Differences in the lipid oxidation-derived VOCs contents, such as hexanal, heptanal, octanal, and nonanal, between Hanwoo and Chikso rump might be due to differences in antioxidant activity. We found that Chikso rump had significantly higher 2,2′-azinobis-3-ethylbenzothiazoline-6-sulfonic acid (ABTS) radical scavenging activity than Hanwoo rump on days 7, 21, and 28 (Supplementary Figure S1). This might help reduce the extent of lipid oxidation, as major lipid oxidation-derived products (hexanal and heptanal) were less abundant in the Chikso rump than in the Hanwoo rump.

**Figure 6:**
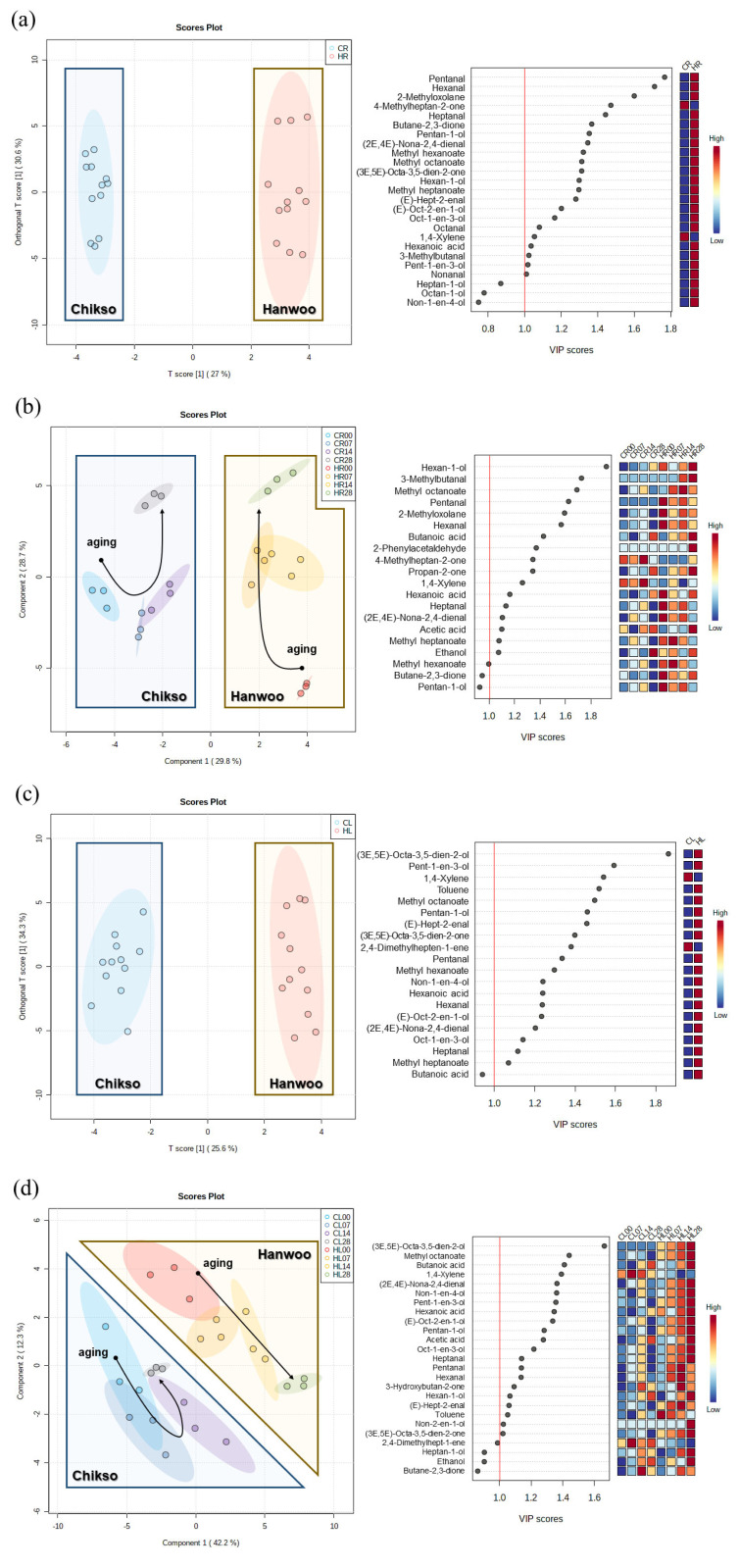
Multivariate analyses and variable importance in projection for each investigation for discriminating volatile organic compound (VOC) profiles between Hanwoo and Chikso beef during wet aging. Orthogonal partial least squares-discriminant analysis (OPLS-DA) between the VOCs in Hanwoo and Chikso beef for the whole wet aging period (days 0 to 28) (a) and partial least squares-discriminant analysis (PLS-DA) between the VOCs in Hanwoo and Chikso rump at each wet aging period (b). Multivariate analyses were performed on beef loins in the same way as beef rumps (c–d). Black arrows in the PLS-DA plot illustrate the direction of aroma pattern change of Hanwoo and Chikso beef during wet aging period. The red-blue color system was used to represent the relative abundance of each VOC in the beef sample. The numbers written in each class indicate the wet aging period of beef samples. CL, Chikso loin; CR, Chikso rump; HL, Hanwoo loin; HR, Hanwoo rump. Multivariate analyses and variable importance in projection for each investigation for discriminating volatile organic compound (VOC) profiles between Hanwoo and Chikso beef during wet aging. Orthogonal partial least squares-discriminant analysis (OPLS-DA) between the VOCs in Hanwoo and Chikso beef for the whole wet aging period (days 0 to 28) (a) and partial least squares-discriminant analysis (PLS-DA) between the VOCs in Hanwoo and Chikso rump at each wet aging period (b). Multivariate analyses were performed on beef loins in the same way as beef rumps (c–d). Black arrows in the PLS-DA plot illustrate the direction of aroma pattern change of Hanwoo and Chikso beef during wet aging period. The red-blue color system was used to represent the relative abundance of each VOC in the beef sample. The numbers written in each class indicate the wet aging period of beef samples. CL, Chikso loin; CR, Chikso rump; HL, Hanwoo loin; HR, Hanwoo rump.

As the wet aging period increased, the alcohol percentage also increased, while that of ketones decreased in the Chikso rump. Ethanol can be generated by microbial fermentation during the aging process [11]. Other compounds, (E)-oct-2-en-1-ol and hexan-1-ol, in the Chikso rump also amplified during aging (p<0.05). However, these compounds have high odor thresholds, and thus contribute less to beef flavor. There was also a significant rise in the aldehyde and ester contents in the Chikso rump until day 14 and then reduced on day 28. On the other hand, volatile fatty acids in the Chikso rump significantly decreased on day 7 but increased thereafter. Wet aging enhances meat flavor by generating flavor precursors and VOCs; however, previous studies have also reported a decrease in major volatile flavor contributors, such as aldehydes, during wet aging. Xu et al [8] reported that the octanal and nonanal concentrations decreased as the wet aging period increased. Similarly, Lee et al [11] reported that the concentration of esters in beef decreased after day 28. In the present study, the total amount of VOCs in both Hanwoo and Chikso rumps decreased after day 28 (Supplementary Table S1). The overall VOC profiles of Hanwoo and Chikso rump were compared using OPLS-DA to identify universal differences between the two breeds (Figure 6a). Two breeds were clearly differentiated which meant that the VOC profiles of Hanwoo and Chikso rump were different. Wei et al [6] reported that the difference in the VOC of different breeds of cattle might be attributed to the fatty acid composition or intramuscular fat content, derived from the genetic difference in lipid metabolism. Furthermore, numerous metabolites including amino acids and nucleotides participate in flavor formation, and therefore the difference in metabolites may also affect the change in VOC profiles of beef [30]. While most VOCs with a VIP>1 were more abundant in the Hanwoo rump than in the Chikso rump, the contents of 4-methylheptan-2-one and 1, 4-xylene were higher in the Chikso rump. During wet aging, the Hanwoo rump showed more remarkable VOC changes than the Chikso rump (Figure 6b).

A total of 41 VOCs were identified in the Hanwoo and Chikso loins (Supplementary Table S2). It is composed of 14 alcohols, 9 aldehydes, 5 esters, 1 furan, 5 hydrocarbons, 4 ketones, and 3 volatile fatty acids. Most VOCs were fewer in the Chikso loin than in the Hanwoo loin. Instead, 1, 4-xylene and 2, 4-dimethylhepten-1-ene were generally more abundant in the Chikso loin throughout the wet aging period than in the Hanwoo loin (Figure 6c).

During wet aging, Hanwoo loin showed a general increase in VOCs; however, Chikso loin only showed a gradual rise in alcohols, but the amounts of aldehydes and esters decreased after 28 days of wet aging (Figure 5), indicating that these two breeds showed different trends in VOC profile changes during wet aging (Figure 6d). Considering the origin of these VOCs, the degree of lipid oxidation might influence the volatile flavor characteristics of Hanwoo and Chikso loins. Chikso loin showed higher antioxidant activities (ABTS radical scavenging activity and ferric reducing antioxidant power) than Hanwoo loin on day 0, which might explain the overall lower quantities of lipid oxidation-derived VOCs in Chikso loin than in Hanwoo loin (Supplementary Figure S1). Overall, VOCs decreased gradually in the Chikso rump during wet aging, while 14 days of wet aging led to abundant VOC content in the Chikso loin.

#### Electronic tongue

**Figure 7:**
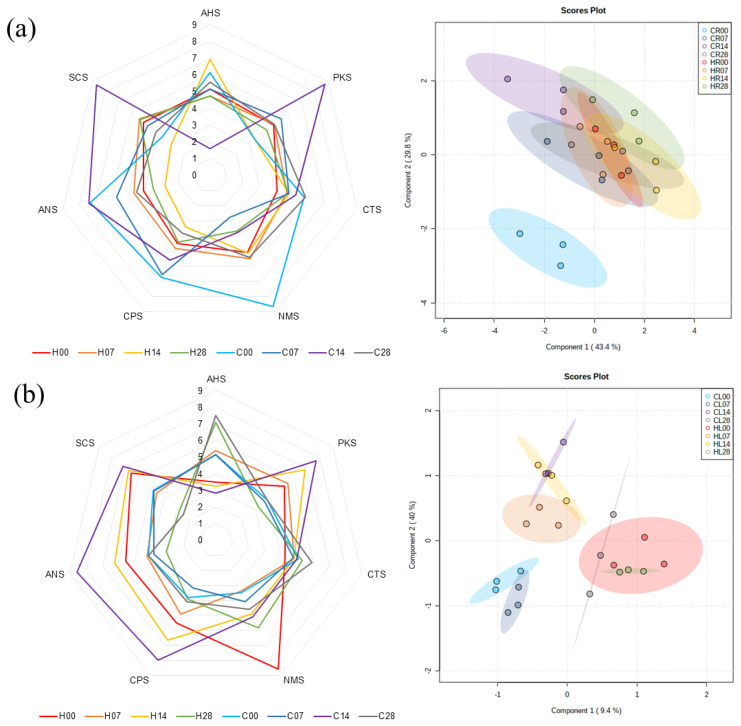
Radar plot and principal component analysis according to the relative taste intensity between Hanwoo and Chikso rump (a) and loin (b) during wet aging. The taste intensity was evaluated using an electronic tongue with seven sensors, where AHS, CTS, NMS, ANS, and SCS respond to sour, salty, umami, sweet, and bitterness, respectively, while PKS and CPS represent universal taste intensity. The numbers written in each class indicate the wet aging period (d) of beef samples. C, Chikso; CL, Chikso loin; CR, Chikso rump; H, Hanwoo; HL, Hanwoo loin; HR, Hanwoo rump.

Electronic tongue analysis was conducted using seven sensors for Hanwoo and Chikso beef on days 0, 7, 14, and 28 (Supplementary Figure S2). The sensors AHS, CTS, NMS, ANS, and SCS respond to sour, salty, umami, sweet, and bitterness, respectively, whereas PKS and CPS represent universal taste intensity. The PLS-DA plot for the electronic tongue results showed that the taste characteristics of the Chikso rump on day 0 were clearly separated from those of Hanwoo rumps on days 0 to 28 and Chikso rumps on days 7 to 28 (Figure 7a). The characteristics of the Chikso rump on day 0 were high values of relative taste intensity for NMS and CPS, indicating a higher intensity for umami taste and universal taste, respectively. In addition, Chiko rump on day 0 showed overall high relative taste intensities, except SCS (bitterness) and PKS (universal), compared to Hanwoo rump on day 0. Umami taste is mainly derived from umami amino acids (aspartic and glutamic acid) and nucleotides (IMP and guanosine 5′-monophosphate [GMP]) [34]. Particularly, the synergistic effect of IMP and glutamic acid could improve the strength of umami taste considerably [8]. Here, the IMP content was the highest in Chikso rump on day 0 of wet aging (Table 2), and a negative correlation between IMP and sensor intensity of NMS was identified (*r* = −0.54; Supplementary Table S4). The lower sensor intensity of NMS indicates a stronger taste intensity for umami taste, and therefore, high content of IMP in Chikso rump on day 0 might contribute to the high umami intensity in electronic tongue analysis.

Additionally, the Chikso rump on day 14 also had different characteristics than the other groups; it showed high values of taste intensity for ANS, SCS, and PKS, which correspond to sweetness, bitterness, and universal taste, respectively. Lee et al [14] reported that dry and wet aging of beef led to the increase of umami taste intensity. However, in our study, umami intensity of Chikso rump decreased considerably from day 0 to 7, although it gradually increased afterwards. Previous studies have reported that the taste characteristics of alanine, glutamine, and glycine impart sweetness, while isoleucine, leucine, phenylalanine, tyrosine, and valine could contribute to bitterness [9]. Moreover, reducing sugars can contribute to the sweetness of the meat, and anserine, carnosine, and nucleosides such as inosine and hypoxanthine provide bitterness [14,30]. In this study, the contents of most of the above-mentioned metabolites were significantly increased during wet aging, which would lead to high intensity for sweetness and bitterness. Especially, considering that umami intensity decreased while bitterness increased dramatically during 14 days of wet aging in Chikso rump, the degradation of umami contributors such as IMP which generated inosine and hypoxanthine at the early phase of wet aging might influence the change of taste attributes of Chikso rump considerably during wet aging. Previous study also noted that PKS is used for the general purpose of taste differentiation in electronic tongues [8], and the relatively high intensity of the Chikso rump on day 14 for the PKS sensor indicates that the universal taste of the Chikso rump on day 14 may be distinguishable from others. In contrast, wet aging for 14 days in Hanwoo rump increased sour taste intensities but lowered other taste intensities. The increase of sourness in wet-aged beef might derive from the accumulation of lactate by the action of lactic acid bacteria [35]. During wet aging for 14 days, lactate content was significantly increased in Hanwoo rump (Table 2).

The PLS-DA plot for taste characteristics of Hanwoo and Chikso loins revealed that loins from the two breeds showed a clear separation on day 0 (Figure 7b). In particular, higher umami intensity was found in Hanwoo loin on day 0 than in Chikso loin. Multivariate analysis also implied that the wet aging period of 14 days for Chikso loin would lead to different taste attributes compared with days 0, 7, or 28. Chikso loin on day 14 showed higher values of ANS, SCS, PKS, and CPS sensor intensities among treatments, indicating a strong intensity of sweetness, bitterness, and universal taste. In contrast, Chikso and Hanwoo loin on day 28 led to high sour attributes, possibly owing to the increased amount of organic acids including acetate, formate or fumarate (p<0.05). This was possibly due to increased alanine, phenylalanine, tyrosine, acetic acid, and formic acid in meat wet-aged for a long period [8,30]. The results suggested that the umami taste intensity was high in Chikso rump and Hanwoo loin on day 0. In contrast, the wet aging process for 14 days was effective in enhancing overall taste intensities and taste-active compounds in the Chikso beef.

## CONCLUSION

Overall, major differences between Hanwoo and Chikso beef on day 0 were observed in the CIE a* value, shear force value, VOCs, and taste attributes. In particular, the higher shear force value in Chikso beef than in Hanwoo beef indicates the need for the wet aging process for meat tenderization. Wet aging decreased the shear force value and increased the MFI of Chikso rump and loin considerably, and it also increased flavor compounds, such as free amino acids and alcoholic VOCs. Furthermore, 14 days of wet aging enhanced the taste intensity of Chikso beef. Therefore, the quality of Chikso beef changed during wet aging, and the wet aging period of 14 days was recommended to improve tenderness, similar to Hanwoo beef, while enhancing its flavor characteristics.

## Figures and Tables

**Table 1:** Changes in the free amino acids, dipeptides and derivatives contents (mg/100 g meat) in Hanwoo and Chikso rump during 28 days of wet aging

Item	Breed	Aging period (d)					SEM^1)^

		0	7	14	21	28	
Alanine	Hanwoo	20.19^c^	21.16^c^^y^	27.93^b^	23.57^bc^^y^	36.35^a^	1.391
	Chikso	21.16^c^	25.66^bc^^x^	33.85^ab^	32.87^ab^^x^	42.44^a^	2.249
	SEM^2)^	1.054	0.675	1.720	2.308	2.762	
Asparagine	Hanwoo	2.78^c^	3.77^c^^y^	6.55^b^^y^	6.52^b^^y^	9.13^a^^y^	0.282
	Chikso	2.68^c^	5.09^bc^^x^	9.59^b^^x^	9.96^b^^x^	16.14^a^^x^	1.137
	SEM^2)^	0.376	0.250	0.296	0.614	1.661	
Glutamate	Hanwoo	6.97^d^	8.60^cd^^y^	12.15^bc^^y^	12.95^b^^y^	17.88^a^^y^	0.815
	Chikso	5.79^c^	10.83^c^^x^	16.56^b^^x^	21.10^b^^x^	34.60^a^^x^	1.138
	SEM^2)^	0.359	0.445	0.617	1.179	1.673	
Glycine	Hanwoo	12.28^b^	13.53^b^^y^	18.50^a^^x^	11.71^b^	11.72^b^^y^	0.791
	Chikso	13.01^b^	14.63^b^^x^	11.78^b^^y^	12.62^b^	21.84^a^^x^	0.635
	SEM^2)^	0.925	0.244	0.574	0.746	0.878	
Isoleucine	Hanwoo	2.37^d^	4.53^cd^^y^	9.17^ab^^y^	8.55^b^^x^	10.94^a^^y^	0.398
	Chikso	2.62^d^	6.87^cd^^x^	11.23^bc^^x^	14.41^b^^x^	25.22^a^^x^	1.374
	SEM^2)^	0.175	0.163	0.447	1.101	1.910	
Leucine	Hanwoo	4.34^d^	8.31^c^^y^	14.80^b^^y^	15.28^b^^y^	19.52^a^^y^	0.594
	Chikso	4.68^e^	13.19^d^^x^	21.50^c^^x^	28.11^b^^x^	45.17^a^^x^	1.358
	SEM^2)^	0.247	0.176	0.483	1.812	1.374	
Methionine	Hanwoo	6.27^c^	8.45^bc^^y^	10.93^b^^y^	10.65^b^^y^	14.11^a^^y^	0.563
	Chikso	6.96^d^	10.53^c^	14.17^b^^x^	17.03^b^^x^	23.23^a^^x^	0.763
	SEM^2)^	0.569	0.368	0.685	0.800	0.825	
Phenylalanine	Hanwoo	3.15^d^	5.62^c^^y^	9.67^b^^y^	10.79^b^^y^	13.08^a^^y^	0.460
	Chikso	3.45^e^	8.08^d^^x^	12.14^c^^x^	16.69^b^^x^	23.83^a^^x^	0.757
	SEM^2)^	0.174	0.180	0.465	1.121	0.651	
Taurine	Hanwoo	27.69^ab^	25.23^ab^	21.83^ab^	20.62^b^	31.23^a^	2.087
	Chikso	24.28	23.98	24.84	21.42	30.88	2.124
	SEM^2)^	2.036	0.806	1.351	2.564	2.995	
Tyrosine	Hanwoo	3.39^y^	5.85^y^	12.88	3.55^y^	6.02^y^	3.628
	Chikso	4.73^d^^x^	8.99^cd^^x^	12.98^bc^	16.82^b^^x^	24.79^a^^x^	1.091
	SEM^2)^	0.320	0.183	5.740	0.939	1.386	
Valine	Hanwoo	3.67^d^	5.85^c^^y^	9.82^b^^y^	9.93^b^^y^	13.96^a^^y^	0.467
	Chikso	3.89^d^	8.79^cd^^x^	14.19^bc^^x^	18.99^ab^^x^	23.60^a^^x^	1.430
	SEM^2)^	0.200	0.103	0.736	1.271	1.856	
Anserine	Hanwoo	67.55^x^	76.90	84.78	78.49	68.61^y^	5.883
	Chikso	56.78^b^^y^	72.34^ab^	70.63^ab^	73.86^a^	85.33^a^^x^	3.543
	SEM^2)^	2.484	3.821	5.874	7.249	3.175	
Carnosine	Hanwoo	193.23^ab^	223.95^ab^^x^	192.54^ab^	239.87^a^	163.67^b^^y^	14.781
	Chikso	145.13^c^	175.81^bc^^y^	163.95^c^	228.56^a^	213.19^ab^^x^	10.537
	SEM^2)^	16.111	9.189	11.695	15.231	10.534	
Creatine	Hanwoo	274.53	258.39	305.93	245.72	278.06	14.346
	Chikso	288.17	278.28	302.93	273.76	312.95	12.322
	SEM^2)^	14.684	6.068	15.437	13.073	15.246	
L-Carnitine	Hanwoo	52.41^bc^^y^	43.47^c^^y^	69.30^ab^^y^	50.70^bc^	72.78^a^	4.034
	Chikso	75.14^abc^^x^	74.52^bc^^x^	106.29^a^^x^	57.75^c^	95.23^ab^	6.799
	SEM^2)^	3.979	1.788	6.919	5.236	7.870	
N,N-Dimethylglycine	Hanwoo	0.69^b^	0.64^b^	0.86^a^	0.67^b^	0.79^ab^	0.035
	Chikso	0.71	0.69	0.84	0.75	0.94	0.080
	SEM^2)^	0.035	0.013	0.036	0.044	0.119	
o-acetylcarnitine	Hanwoo	26.06^y^	21.43^y^	22.68^x^	21.86	24.44^x^	1.396
	Chikso	36.08^a^^x^	29.35^a^^x^	5.14^c^^y^	21.17^b^	5.32^c^^y^	1.488
	SEM^2)^	2.108	1.418	0.404	1.499	1.243	
Total	Hanwoo	707.58	735.68	830.32	771.42	792.29^y^	38.019
	Chikso	695.24^b^	767.65^b^	832.60^b^	865.88^ab^	1,024.69^a^^x^	38.448
	SEM^2)^	34.460	14.599	39.126	49.877	43.476	

1) Standard error of the mean (n = 15),

2) (n = 6).

a–e Different letters within the same row indicate significant differences (p<0.05).

x,y Different letters within the same column indicate significant differences (p<0.05).

**Table 2:** Changes in the nucleotides and nucleosides, organic acids, ethanol and niacinamide contents (mg/100 g meat) in Hanwoo and Chikso rump during 28 days of wet aging

Item	Breed	Aging period (d)					SEM^1)^

		0	7	14	21	28	
Nucleotide and nucleosides							
Hypoxanthine	Hanwoo	21.94^c^	32.69^bc^	37.03^ab^	41.45^ab^	48.59^a^	2.566
	Chikso	17.83^c^	29.79^b^	42.88^a^	37.73^ab^	42.22^a^	2.316
	SEM^2)^	1.862	2.664	2.099	2.440	2.992	
IMP	Hanwoo	71.08^a^	48.22^b^	49.99^b^	32.41^bc^	17.99^c^^y^	4.457
	Chikso	83.54^a^	58.33^b^	45.44^c^	30.58^d^	30.06^d^^x^	1.825
	SEM^2)^	3.854	5.789	1.876	1.961	1.505	
Inosine	Hanwoo	14.56^ab^	14.81^ab^^y^	17.78^a^	13.21^b^	11.56^b^^y^	0.862
	Chikso	16.49^b^	19.07^ab^^x^	18.19^ab^	17.67^ab^	19.68^a^^x^	0.662
	SEM^2)^	0.630	0.611	0.726	1.229	0.384	
Uridine	Hanwoo	1.26^c^	1.54^bc^	2.14^ab^	1.82^ab^	1.99^a^^y^	0.088
	Chikso	1.05^c^	1.46^bc^	2.05^ab^	2.54^a^	2.70^a^^x^	0.181
	SEM^2)^	0.083	0.044	0.147	0.241	0.113	
Subtotal	Hanwoo	107.57^a^	95.72^ab^	104.81^a^	87.08^ab^	78.13^bc^	4.805
	Chikso	118.17^a^	107.18^ab^	106.51^ab^	85.98^c^	91.96^bc^	4.124
	SEM^2)^	5.586	3.906	3.789	5.066	3.709	
Organic acids							
Acetate	Hanwoo	4.80^c^	5.40^c^	9.45^b^^y^	8.22^b^	16.87^a^	0.530
	Chikso	4.49^b^	5.93^b^	16.82^a^^x^	6.78^b^	20.89^a^	0.896
	SEM^2)^	0.276	0.315	0.242	0.533	1.480	
Formate	Hanwoo	0.31^c^	0.33^c^	0.46^c^^y^	2.02^b^^x^	3.95^a^^x^	0.233
	Chikso	0.28^c^	0.32^c^	1.32^b^^x^	0.34^c^^y^	1.90^a^^y^	0.099
	SEM^2)^	0.033	0.014	0.101	0.058	0.381	
Fumarate	Hanwoo	1.61^b^^y^	2.73^ab^	6.38^a^	3.61^ab^^y^	6.56^a^	0.840
	Chikso	3.23^x^	3.99	5.87	5.29^x^	6.57	0.798
	SEM^2)^	0.186	1.381	0.457	0.377	1.031	
Lactate	Hanwoo	476.11^b^	454.23^b^^y^	593.35^a^	454.06^b^	539.37^ab^^y^	21.588
	Chikso	506.60^bc^	524.81^bc^^x^	591.92^ab^	493.73^c^	620.41^a^^x^	20.118
	SEM^2)^	19.887	13.668	20.418	27.773	20.159	
Subtotal	Hanwoo	482.82^bc^	462.69^c^^y^	609.64^a^	467.91^bc^	566.75^ab^	21.933
	Chikso	514.60^c^	535.04^bc^^x^	615.92^ab^	506.15^c^	649.77^a^	21.008
	SEM^2)^	20.101	14.669	20.315	28.472	21.527	
Others							
Ethanol	Hanwoo	0.56^y^	0.56^y^	0.93	1.15	1.88	0.311
	Chikso	1.96^a^^x^	1.05^bc^^x^	0.95^c^	1.72^ab^	1.96^a^	0.155
	SEM^2)^	0.163	0.084	0.133	0.157	0.476	
Niacinamide	Hanwoo	3.37^b^	3.68^ab^^y^	4.32^a^	3.72^ab^	3.47^ab^^y^	0.189
	Chikso	3.41^b^	4.39^ab^^x^	4.63^a^	4.76^a^	5.31^a^^x^	0.242
	SEM^2)^	0.154	0.160	0.146	0.284	0.292	

IMP, inosine 5′-monophosphate.

1) Standard error of the mean (n = 15),

2) (n = 6).

a–d Different letters within the same row indicate significant differences (p<0.05).

x,y Different letters within the same column indicate significant differences (p<0.05).

**Table 3:** Changes in the free amino acids, dipeptides and derivatives contents (mg/100 g meat) in Hanwoo and Chikso loin during 28 days of wet aging

Item	Breed	Aging period (d)					SEM^1)^

		0	7	14	21	28	
Alanine	Hanwoo	19.07^c^	21.34^bc^^y^	27.31^abc^	29.55^ab^^y^	36.89^a^	2.110
	Chikso	22.80^c^	28.33^bc^^x^	28.12^bc^	37.09^a^^x^	33.37^ab^	1.490
	SEM^2)^	1.681	0.695	1.095	1.113	3.306	
Asparagine	Hanwoo	2.70^c^	2.83^y^	4.37^bc^	5.48^b^	8.73^a^	0.433
	Chikso	3.10^d^	4.91^cd^^x^	5.37^bc^	7.55^ab^	8.46^a^	0.486
	SEM^2)^	0.374	0.132	0.405	0.663	0.547	
Glutamate	Hanwoo	7.73^c^	7.82^c^	11.04^bc^	11.87^b^^y^	17.05^a^	0.745
	Chikso	6.50^c^	8.89^bc^	10.95^b^	16.31^a^^x^	17.08^a^	0.861
	SEM^2)^	0.753	0.741	0.425	1.123	0.827	
Glycine	Hanwoo	9.58	10.12	10.13	10.95	10.79	1.006
	Chikso	9.06	9.26	10.85	10.82	7.32	0.870
	SEM^2)^	0.610	0.790	0.878	0.866	1.381	
Isoleucine	Hanwoo	1.77^d^	2.45^cd^^y^	4.17^bc^	5.56^ab^	7.21^a^	0.388
	Chikso	1.85^c^	4.37^bc^^x^	4.80^b^	8.12^a^	7.86^a^	0.620
	SEM^2)^	0.203	0.130	0.377	0.708	0.797	
Leucine	Hanwoo	3.21^c^	4.59^c^^y^	7.75^b^	10.11^a^	12.22^a^	0.485
	Chikso	3.27^b^	7.89^b^^x^	9.03^ab^	14.47^a^	14.75^a^	1.245
	SEM^2)^	0.372	0.238	0.369	1.269	1.590	
Methionine	Hanwoo	5.62^b^	6.16^b^	8.95^b^	8.07^b^	13.93^a^	1.048
	Chikso	7.23	8.36	8.75	12.22	11.12	1.533
	SEM^2)^	0.618	0.966	1.806	1.660	1.137	
Phenylalanine	Hanwoo	1.96^c^	2.85^c^^y^	4.88^b^	6.59^a^	7.70^a^	0.264
	Chikso	2.04^b^	5.18^b^^x^	5.56^b^	9.73^a^	9.65^a^	0.767
	SEM^2)^	0.214	0.167	0.220	0.835	0.909	
Taurine	Hanwoo	25.39	27.21	28.21	31.48^x^	19.18	3.098
	Chikso	24.43	28.61	21.93	23.83^y^	26.96	2.337
	SEM^2)^	3.918	3.013	2.072	0.829	2.868	
Tyrosine	Hanwoo	2.17	3.09^y^	2.59	2.35^y^	2.39^y^	0.229
	Chikso	2.10^d^	5.05^bc^^x^	3.84^cd^	5.94^b^^x^	9.58^a^^x^	0.446
	SEM^2)^	0.240	0.243	0.454	0.520	0.191	
Valine	Hanwoo	2.76^d^	3.59^d^^y^	5.74^c^	7.82^b^	11.66^a^	0.344
	Chikso	2.72^c^	5.93^c^^x^	7.31^bc^	11.18^ab^	14.05^a^	1.124
	SEM^2)^	0.278	0.080	0.497	0.982	1.469	
Anserine	Hanwoo	30.78	23.46^y^	40.52^x^	44.98^x^	27.01	4.681
	Chikso	38.42	37.51^x^	27.26^y^	32.64^y^	21.38	3.815
	SEM^2)^	7.514	2.737	3.275	3.023	2.708	
Carnosine	Hanwoo	113.62	82.50^y^	125.19	109.39	102.65	14.552
	Chikso	140.56	157.95^x^	93.98	127.33	98.79	14.353
	SEM^2)^	15.561	9.499	11.573	19.591	13.939	
Creatine	Hanwoo	196.29	183.25^y^	215.77^x^	194.61	180.51	15.926
	Chikso	212.32^abc^	241.85^a^^x^	171.57^bc^^y^	218.37^ab^	152.20^c^	13.072
	SEM^2)^	18.660	3.076	8.879	12.089	21.879	
L-Carnitine	Hanwoo	48.35	49.04^y^	62.74^y^	51.19^y^	64.48	5.276
	Chikso	74.16	71.68^x^	90.08^x^	82.25^x^	86.03	4.871
	SEM^2)^	7.126	2.893	2.418	4.325	6.724	
N,N-Dimethylglycine	Hanwoo	0.53	0.49^y^	0.63^x^	0.56	0.67	0.041
	Chikso	0.55	0.62^x^	0.50^y^	0.66	0.63	0.042
	SEM^2)^	0.059	0.014	0.026	0.024	0.061	
o-acetylcarnitine	Hanwoo	20.37^a^	20.73^a^^y^	22.47^a^^x^	21.93^a^	2.01^b^^y^	1.990
	Chikso	24.32^a^	30.07^a^^x^	3.19^b^^y^	29.89^a^	3.43^b^^x^	2.317
	SEM^2)^	2.885	1.860	2.211	2.556	0.338	
Total	Hanwoo	491.89	451.52^y^	582.46	552.49	525.08	40.922
	Chikso	575.43	656.44^x^	503.08	648.41	522.66	35.414
	SEM^2)^	53.399	5.492	21.354	35.547	52.160	

1) Standard error of the mean (n = 15),

2) (n = 6).

a–d Different letters within the same row indicate significant differences (p<0.05).

x,y Different letters within the same column indicate significant differences (p<0.05).

**Table 4:** Changes in the nucleotides and nucleosides, organic acids, ethanol and niacinamide contents (mg/100 g meat) in Hanwoo and Chikso loin during 28 days of wet aging

Item	Breed	Aging period (d)					SEM^1)^

		0	7	14	21	28	
Nucleotide and nucleosides							
Hypoxanthine	Hanwoo	17.25^b^	23.75^b^^y^	33.39^a^^x^	35.48^a^	37.57^a^	1.778
	Chikso	15.76^c^	28.64^b^^x^	23.29^bc^^y^	39.39^a^	26.98^b^	1.978
	SEM^2)^	1.428	0.318	1.124	1.137	3.604	
IMP	Hanwoo	32.33^a^	13.15^b^^y^	7.67^b^	5.47^b^	4.54^b^	2.752
	Chikso	49.42^a^	38.33^a^^x^	6.90^b^	9.29^b^	4.56^b^	3.359
	SEM^2)^	4.685	4.161	0.920	2.601	0.527	
Inosine	Hanwoo	8.87^a^	7.02^a^^y^	7.50^a^^x^	6.27^a^	1.46^b^^y^	0.649
	Chikso	9.84^a^	10.32^a^^x^	4.08^b^^y^	5.59^b^	3.89^b^^x^	0.824
	SEM^2)^	1.098	0.674	0.387	0.889	0.387	
Uridine	Hanwoo	1.13	1.26^y^	1.46	1.40	1.87	0.227
	Chikso	0.88^b^	1.52^a^^x^	1.19^ab^	1.28^ab^	1.25^ab^	0.122
	SEM^2)^	0.108	0.055	0.075	0.088	0.372	
Subtotal	Hanwoo	58.45	43.92^y^	48.56^x^	47.21	43.58	4.147
	Chikso	75.03^a^	77.29^a^^x^	34.27^b^^y^	54.28^ab^	35.43^b^	5.085
	SEM^2)^	6.929	4.857	1.558	4.601	3.528	
Organic acids							
Acetate	Hanwoo	3.70^b^	4.07^b^	6.89^b^^y^	12.43^b^	29.85^a^	1.887
	Chikso	4.31^c^	4.07^c^	12.87^b^^x^	14.87^b^	21.95^a^	1.305
	SEM^2)^	0.743	0.272	0.548	0.736	3.419	
Formate	Hanwoo	0.26^c^	0.24^c^^y^	2.76^c^	8.19^b^	17.73^a^^x^	1.056
	Chikso	0.31^b^	0.32^b^^x^	3.84^b^	12.71^a^	8.33^a^^y^	0.956
	SEM^2)^	0.044	0.015	0.432	1.743	1.360	
Fumarate	Hanwoo	0.61^b^^y^	1.38b	1.22^b^	4.24^a^	4.92^a^	0.513
	Chikso	0.99^b^^x^	1.29^b^	1.33^b^	3.69^a^	3.34^a^	0.197
	SEM^2)^	0.047	0.396	0.130	0.388	0.655	
Lactate	Hanwoo	313.32	302.02^y^	345.15^x^	357.84	350.02	29.294
	Chikso	337.30	385.98^x^	288.36^y^	366.71	328.70	22.912
	SEM^2)^	18.764	17.058	9.331	12.308	50.756	
Subtotal	Hanwoo	317.89	307.72^y^	356.02^x^	382.70	402.52	32.079
	Chikso	342.92	391.67^x^	306.39^y^	397.98	362.32	24.079
	SEM^2)^	19.167	17.482	9.880	12.774	55.573	
Others							
Ethanol	Hanwoo	0.52^b^^y^	0.39^b^	0.49^b^	0.86^b^	1.51^a^^x^	0.134
	Chikso	1.00^x^	0.57	0.55	1.07	0.69^y^	0.122
	SEM^2)^	0.088	0.047	0.089	0.167	0.191	
Niacinamide	Hanwoo	2.06^a^	2.29^a^^y^	2.70^a^	2.20^a^	0.65^b^	0.198
	Chikso	2.77^ab^	3.43^a^^x^	2.09^bc^	2.34^b^	1.34^c^	0.187
	SEM^2)^	0.188	0.162	0.159	0.215	0.230	

IMP, inosine 5′-monophosphate.

1) Standard error of the mean (n = 15),

2) (n = 6).

a–c Different letters within the same row indicate significant differences (p<0.05).

x,y Different letters within the same column indicate significant differences (p<0.05).

## SUPPLEMENTARY MATERIAL

**Supplementary Figure S1.** Antioxidant activities of Hanwoo and Chikso beef cuts during aging period.

**Supplementary Figure S2.** Sensor value of seven electronic tongue sensors for Hanwoo and Chikso rump (a) and loin (b) during aging, respectively.

**Supplementary Table S1.** Volatile flavor compounds (area unit ×10^6^) in Hanwoo and Chikso rump during 28 days of aging

**Supplementary Table S2.** Volatile flavor compounds (area unit ×10^6^) in Hanwoo and Chikso loin during 28 days of aging

**Supplementary Table S3.** Pearson correlation coefficient (n = 30) between meat quality traits and metabolites in Hanwoo and Chikso beef cuts

**Supplementary Table S4.** Pearson correlation coefficient (n = 24) between metabolites of Hanwoo and Chikso beef cuts and their sensor values of electronic tongue
