# Supplementary material for: Evaluation of the physicochemical, metabolomic, and sensory characteristics of Chikso and Hanwoo beef during wet aging
Source: Anim Biosci. 2023 Feb 26;36(7):1101–19. doi: 10.5713/ab.23.0001 (PMC10330996; doi:10.5713/ab.23.0001)
Supplement: Supplementary file 1 [file ab-23-0001-Supplementary-Fig-1.pdf]

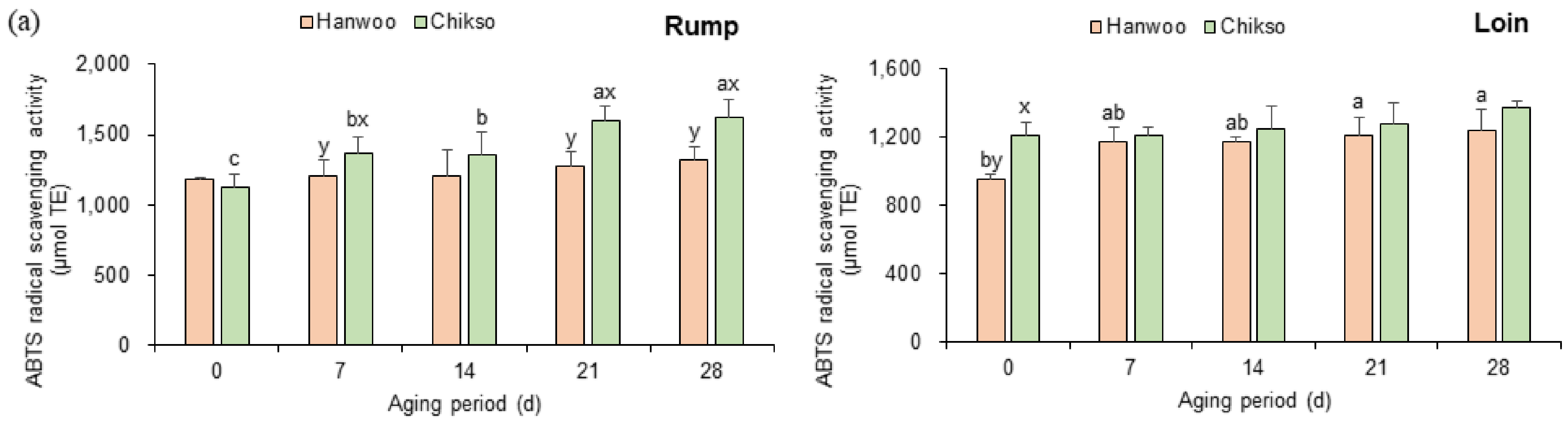
(a)
Hanwoo
Chikso
Rump
ABTS radical scavenging activity (µmol TE)
2,000
1,500
1,000
500
0
c
y
bx
b
y
ax
y
ax
0
7
14
21
28
Aging period (d)
Hanwoo
Chikso
Loin
ABTS radical scavenging activity (µmol TE)
1,600
1,200
800
400
0
by
x
ab
ab
a
a
0
7
14
21
28
Aging period (d)

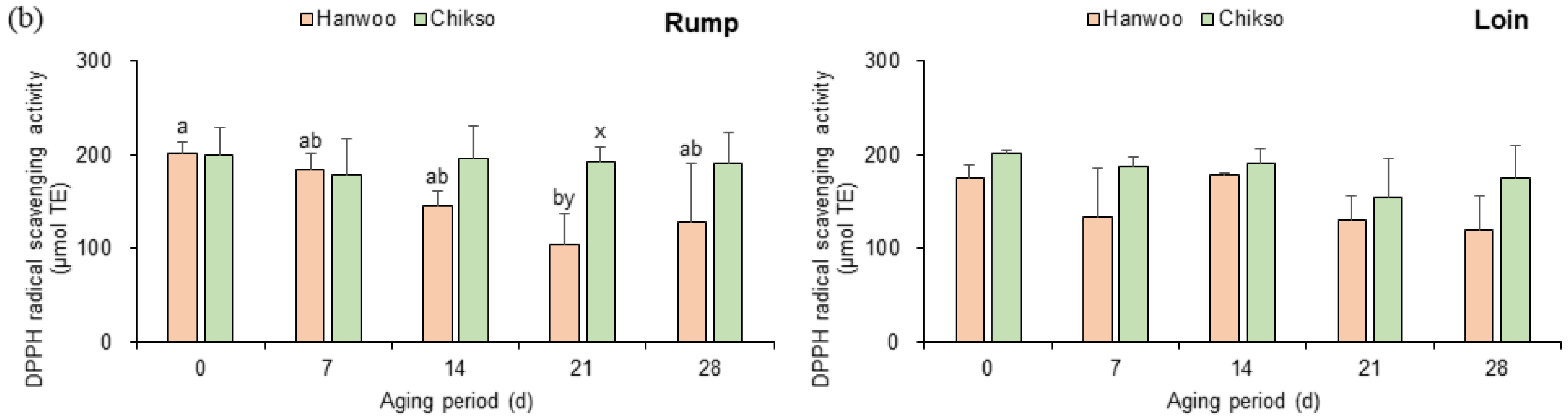
(b)
Hanwoo
Chikso
Rump
DPPH radical scavenging activity (µmol TE)
300
200
100
0
a
ab
ab
by
x
ab
0
7
14
21
28
Aging period (d)
Hanwoo
Chikso
Loin
DPPH radical scavenging activity (µmol TE)
300
200
100
0
0
7
14
21
28
Aging period (d)

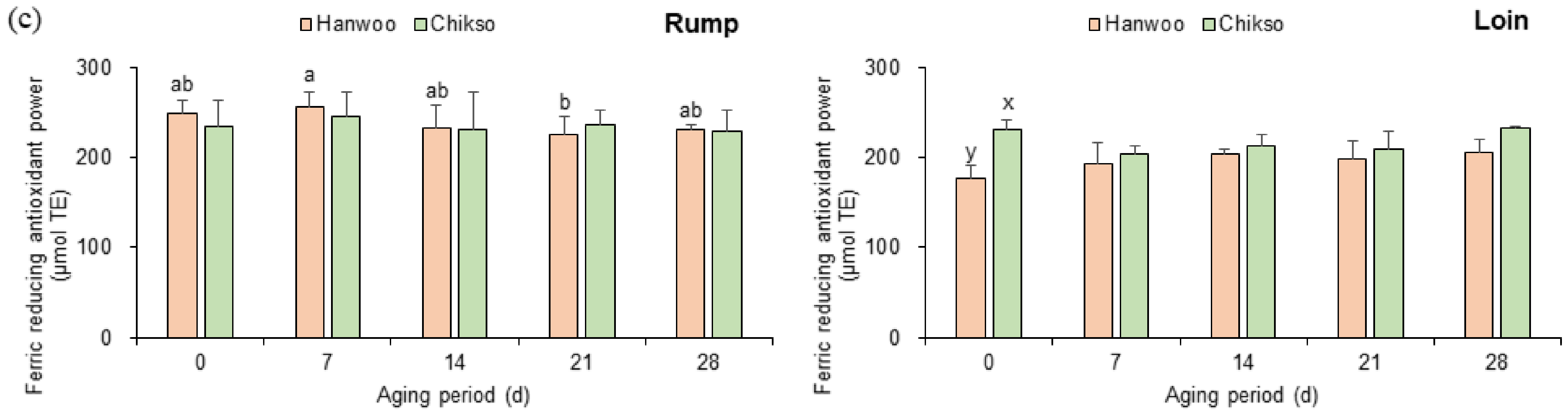


**Figure S1.** Antioxidant activities of Hanwoo and Chikso beef cuts during aging period. The antioxidant activities were expressed as μmol trolox equivalents (TE). [a-c] Different letters within the same breed indicate significant differences ($p < 0.05$). [x,y] Different letters within the same aging period indicate significant differences ($p < 0.05$). ABTS, 2,2′-Azinobis-3-ethylbenzothiazoline-6-sulfonic acid; DPPH, 2,2-Diphenyl-1-picrylhydrazyl.
