# Supplementary material for: Evaluation of the physicochemical, metabolomic, and sensory characteristics of Chikso and Hanwoo beef during wet aging
Source: Anim Biosci. 2023 Feb 26;36(7):1101–19. doi: 10.5713/ab.23.0001 (PMC10330996; doi:10.5713/ab.23.0001)
Supplement: Supplementary file 2 [file ab-23-0001-Supplementary-Fig-2.pdf]

(a)

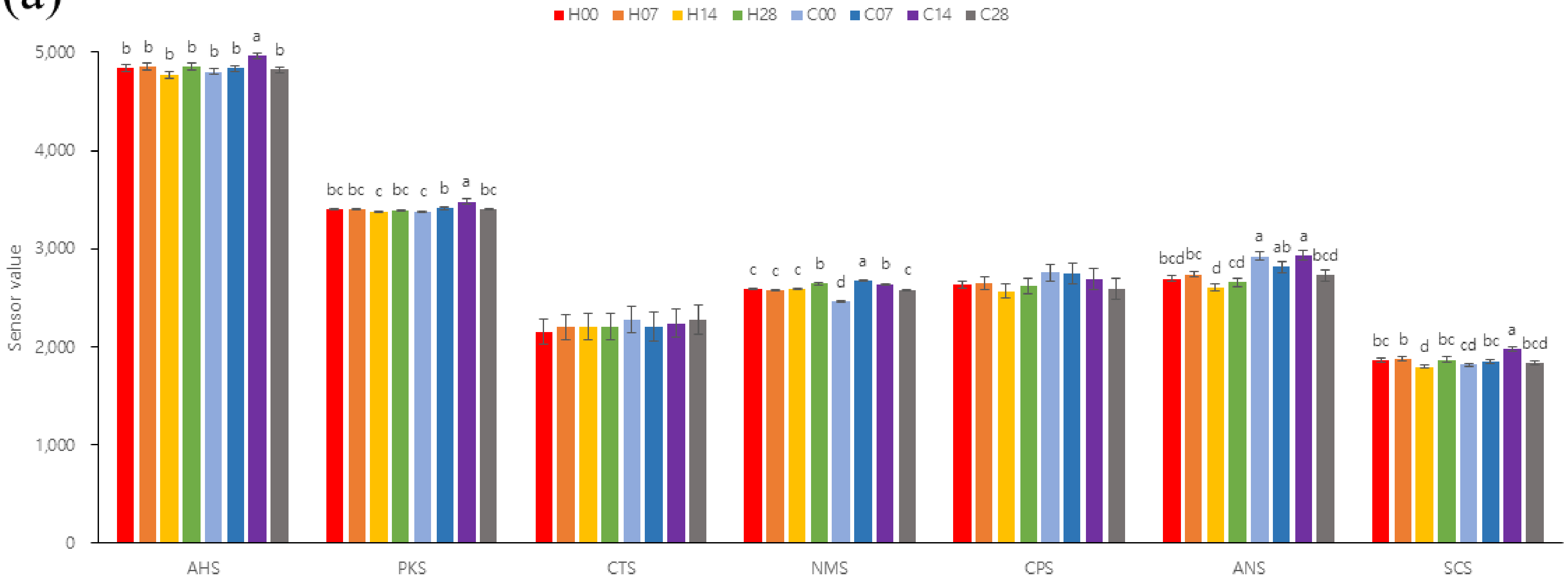
H00 H07 H14 H28 C00 C07 C14 C28
Sensor value
5,000
4,000
3,000
2,000
1,000
0
AHS
PKS
CTS
NMS
CPS
ANS
SCS

(b)

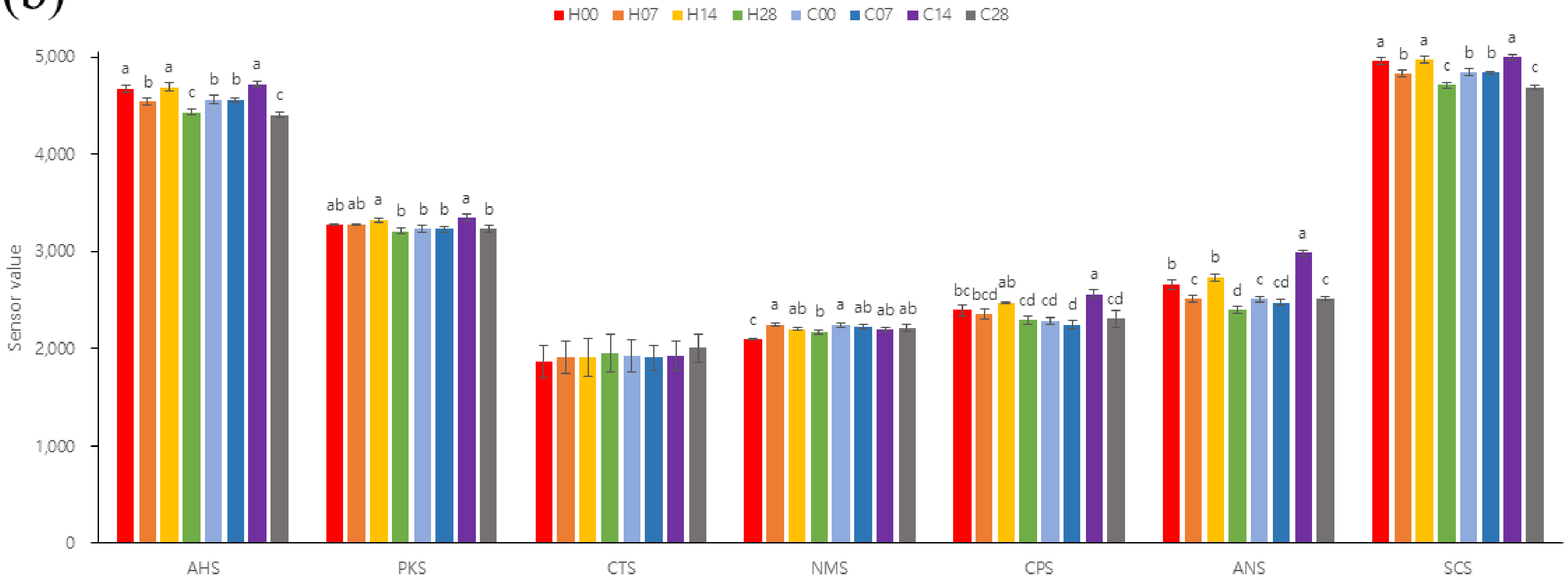

Figure S2. Sensor value of seven electronic tongue sensors for Hanwoo and Chikso rump (a) and loin (b) during aging, respectively. The numbers written in each class indicate the aging period (d) of beef samples. AHS, CTS, NMS, ANS, and SCS respond to sour, salty, umami, sweet, and bitterness, respectively, while PKS and CPS represent universal taste intensity. For AHS and NMS sensors, lower value indicates higher taste intensity. [a-d] Different letters within treatments indicate significant differences ($p < 0.05$). C, Chikso; H, Hanwoo.
