# Supplementary material for: Evaluation of the physicochemical, metabolomic, and sensory characteristics of Chikso and Hanwoo beef during wet aging
Source: Anim Biosci. 2023 Feb 26;36(7):1101–19. doi: 10.5713/ab.23.0001 (PMC10330996; doi:10.5713/ab.23.0001)
Supplement: Supplementary file 3 [file ab-23-0001-Supplementary-Table-1.pdf]

Supplementary data

Evaluation of the physicochemical, metabolomic and sensory characteristics of Chikso and Hanwoo beef during wet aging

Dongheon *Lee*[1], Hye-Jin *Kim*[1], Azfar *Ismail*[1], Sung-Su *Kim*[1], Dong-Gyun *Yim*[1], and Cheorun *Jo*[1,2,*]

[*]**Corresponding Author: Cheorun Jo**

Tel: +82-2-880-4804, Fax: +82-2-880-2271, E-mail: cheorun@snu.ac.kr

[1] Department of Agricultural Biotechnology, Center for Food and Bioconvergence, and Research Institute of Agriculture and Life Science, Seoul National University, Seoul 08826, Republic of Korea

[2] Institute of Green Bio Science and Technology, Seoul National University, Pyeongchang 25354, Republic of Korea

MATERIALS AND METHODS

Antioxidant activity

Sample preparation for the determination of antioxidant activity

A meat sample (4 g) was homogenized with 20 mL of distilled water, and filtered through filter paper (Whatman No. 4). Then, 4 mL of chloroform was added to the filtrates and vortexed. The top layer was extracted and lyophilized, and stored at -20°C for further analyses.

2,2'-Azinobis-3-ethylbenzothiazoline-6-sulfonic acid (ABTS) radical scavenging activity assay

The ABTS radical scavenging activity assay was performed according to Re et al. [1]. A 7 mM ABTS+ solution and 2.45 mM potassium persulfate were mixed at a ratio of 1:1 (v/v), and incubated at 25°C for 12 to 16 h in the dark. The mixture was diluted with ethanol until its absorbance at 735 nm reached 0.70 ± 0.02. Then, 50 μL of sample and 950 μL of ABTS solution were reacted at 30°C for 30 min, followed by measuring the absorbance of the sample at 735 nm using a UV/Vis spectrophotometer (Molecular devices M2e, Molecular Devices). The result was expressed as μmol trolox equivalents (TE)/mg dry matter using trolox as standard.

2,2-Diphenyl-1-picrylhydrazyl (DPPH) radical scavenging activity assay

The DPPH radical scavenging activity assay was conducted using the method described by Blois [2]. Briefly, 100 μL of the sample was reacted with 100 μL of 0.2 mM DPPH in methanol at 25°C for 30 min in the dark. The absorbance of the sample was measured at 517 nm using a UV/Vis spectrophotometer (Molecular devices M2e, Molecular Devices). Trolox was used as a standard and the result was expressed as μmol TE/mg dry matter.

Ferric reducing antioxidant power (FRAP) assay

The FRAP assay was performed according to Benzie and Strain [3]. FRAP solution was prepared by mixing of 300 mM acetate buffer (pH 3.6), 10 mM of 2, 4, 6-tripyridyl-S-triazine in 40 mM HCl, and 20 mM ferric chloride hexahydrate at a ratio of 10:1:1 (v/v/v), respectively. Then, 175 μL of FRAP solution was added to 25 μL of sample and stored at 37°C for 30 min. The absorbance of the sample was measured at 590 nm using a UV/Vis spectrophotometer (Molecular devices M2e, Molecular Devices). The result was expressed as μmol TE/mg dry matter using trolox as standard.

REFERENCES

1. Benzie IF, Strain JJ. The ferric reducing ability of plasma (FRAP) as a measure of "antioxidant power": the FRAP assay. Anal Biochem 1996;239:70-76. https://doi.org/10.1006/abio.1996.0292
2. Blois MS. Antioxidant determinations by the use of a stable free radical. Nature 1958;181:1199-1200. https://doi.org/10.1038/1811199a0
3. Re R, Pellegrini N, Proteggente A, Pannala A, Yang M, Rice-Evans C. Antioxidant activity applying an improved ABTS radical cation decolorization assay. Free Radic Biol Med 1999;26:1231-1237. https://doi.org/10.1016/S0891-5849(98)00315-3

Table S1. Volatile flavor compounds (area unit × 10^6) in Hanwoo and Chikso rump during 28 days of aging.

Compound	LRI	Breed	Aging period (d)				SEM[1)]	Aroma description
			0	7	14	28		
Alcohols								
(E)-Oct-2-en-1-ol	1626	Hanwoo	2.33^{ax}	0.33^{bcx}	0.45^{bx}	0.21^{c}	0.049	Citrus, green, soapy
		Chikso	0.13^{by}	0.22^{ay}	0.27^{ay}	-	0.013	
		SEM[2)]	0.052	0.023	0.042	0.004		
2-Methylbutan-2-ol	1020	Hanwoo	0.27	0.37	0.07^{y}	0.22	0.081	Pungent
		Chikso	0.28^{ab}	0.20^{ab}	0.40^{ax}	0.12^{b}	0.058	
		SEM[2)]	0.104	0.070	0.048	0.040		
2-Methylpropan-2-ol	884	Hanwoo	2.10	2.54	0.53	1.51	0.820	Camphor
		Chikso	1.87	1.42	2.92	0.77	0.660	
		SEM[2)]	0.984	0.743	0.729	0.403		
3-Methylbutan-1-ol	1217	Hanwoo	-	-	-	0.85	0.014	Fruity, malty, oily
		Chikso	0.05^{b}	-	-	0.20^{a}	0.005	

		SEM[2)]	0.006	0.000	0.000	0.020		
Ethanol	927	Hanwoo	3.56[cx]	7.10[bx]	1.78[dx]	14.91[ay]	0.062	Alcoholic, ethereal, sweet
		Chikso	1.21[cy]	3.28[by]	1.34[cy]	26.43[ax]	0.156	
		SEM[2)]	0.073	0.077	0.043	0.208		
Heptan-1-ol	1466	Hanwoo	1.94[ax]	0.35[b]	0.57[b]	0.67[bx]	0.076	Herbal, musty, sweet
		Chikso	0.31[by]	0.66[a]	0.55[ab]	0.28[by]	0.072	
		SEM[2)]	0.081	0.078	0.079	0.055		
Hexan-1-ol	1363	Hanwoo	8.91[bx]	1.75[c]	4.63[bc]	15.84[ax]	0.963	Ethereal, fruity, sweet
		Chikso	0.50[cy]	1.17[bc]	1.38[b]	4.00[ay]	0.161	
		SEM[2)]	0.248	0.186	0.880	1.018		
Non-1-en-4-ol	1671	Hanwoo	0.54	-	-	-	0.030	Grassy, honey[(2)]
		Chikso	-	-	-	-	0.000	
		SEM[2)]	0.043	0.000	0.000	0.000		

Oct-1-en-3-ol	1460	Hanwoo	49.75[ax]	6.40[bcx]	8.83[b]	3.73[cx]	1.081	Fishy, fatty, mushroom(3)
		Chikso	2.59[cy]	4.30[by]	5.98[a]	0.95[dy]	0.281	
		SEM2)	1.153	0.523	0.933	0.159		
Octan-1-ol	1566	Hanwoo	1.57[ax]	0.61[b]	0.34[c]	0.38[cx]	0.033	Waxy, green, mushroom
		Chikso	0.29[bcy]	0.68[a]	0.45[b]	0.19[cy]	0.045	
		SEM2)	0.045	0.045	0.043	0.018		
Pent-1-en-3-ol	1170	Hanwoo	2.63[ax]	0.90[bx]	1.06[b]	0.53[cx]	0.074	Buttery, green, fruity
		Chikso	0.74[by]	0.47[cy]	0.96[a]	0.36[dy]	0.022	
		SEM2)	0.065	0.044	0.075	0.013		
Pentan-1-ol	1260	Hanwoo	22.28[ax]	4.95[bx]	7.34[bx]	2.35[cx]	0.538	Balsamic, oily, sweet
		Chikso	1.27[by]	2.53[ay]	2.99[ay]	0.60[by]	0.187	
		SEM2)	0.141	0.578	0.541	0.027		

Subtotal		Hanwoo	96.83[ax]	25.31[cx]	25.75[c]	41.20[bx]	2.876	
		Chikso	9.24[cy]	14.91[by]	17.22[b]	33.94[ay]	1.245	
		SEM[2)]	2.203	2.145	2.742	1.634		
Aldehydes								
(2E,4E)-Nona-2,4-dienal	1730	Hanwoo	1.19[a]	0.19[cx]	0.32[bx]	0.05[d]	0.027	Fatty, green, nutty
		Chikso	-	0.10[ay]	0.06[by]	-	0.006	
		SEM[2)]	0.019	0.016	0.028	0.008		
(E)-Hept-2-enal	1349	Hanwoo	3.07[a]	0.40[cx]	0.86[b]	-	0.092	Fatty, green, almond
		Chikso	-	0.08[y]	-	-	0.009	
		SEM[2)]	0.125	0.032	0.018	0.000		
(E)-Oct-2-enal	1456	Hanwoo	0.13	-	-	-	0.013	Fatty, green, nutty
		Chikso	-	-	-	-	0.000	
		SEM[2)]	0.019	0.000	0.000	0.000		
2-Phenylacetaldehyde	1676	Hanwoo	-	-	-	2.85	0.072	Cocoa, floral, green
		Chikso	-	-	-	-	0.000	

		SEM[2)]	0.000	0.000	0.000	0.000		
3-Methylbutanal	911	Hanwoo	-	-	0.09[b]	1.69[a]	0.058	Fatty, malty, ethereal
		Chikso	-	-	-	-	0.000	
		SEM[2)]	0.000	0.000	0.016	0.081		
Heptanal	1203	Hanwoo	16.12[ax]	2.84[cx]	4.90[bx]	0.92[cx]	0.452	Fatty, green, rancid
		Chikso	0.25[by]	0.97[ay]	1.02[ay]	0.17[by]	0.054	
		SEM[2)]	0.228	0.236	0.552	0.045		
Hexanal	1100	Hanwoo	261.96[ax]	36.22[cx]	75.72[bx]	15.86[cx]	4.993	Fatty, green, fruity
		Chikso	0.84[by]	3.19[ay]	3.53[ay]	0.57[by]	0.330	
		SEM[2)]	4.900	4.371	2.280	1.330		
Nonanal	1414	Hanwoo	7.27[ax]	2.57[b]	1.83[c]	1.02[dx]	0.116	Fatty, green, fishy
		Chikso	0.37[cy]	2.52[a]	1.81[b]	0.22[cy]	0.104	
		SEM[2)]	0.19	0.141	0.111	0.045		
Octanal	1308	Hanwoo	1.77[a]	0.41[bc]	0.49[b]	0.19[c]	0.053	Fatty, green, citrus
		Chikso	-	0.45	0.38	-	0.024	

		SEM[2)]	0.065	0.026	0.042	0.008		
Pentanal	996	Hanwoo	8.49[a]	1.25[c]	2.64[b]	0.61[d]	0.134	Malty, nutty, fruity
		Chikso	-	-	-	-	0.000	
		SEM[2)]	0.087	0.139	0.069	0.067		
Subtotal		Hanwoo	300.00[ax]	43.88[cx]	86.86[bx]	23.19[cx]	5.324	
		Chikso	1.46[by]	7.32[ay]	6.81[ay]	0.96[by]	0.337	
		SEM[2)]	4.752	4.830	3.054	1.297		
Esters								
Ethyl butanoate	1050	Hanwoo	-	-	-	0.16[y]	0.006	Fruity
		Chikso	-	-	-	0.33[x]	0.004	
		SEM[2)]	0.000	0.000	0.000	0.011		
Methyl 2-hydroxypropanoate	1337	Hanwoo	1.32[by]	2.60[a]	2.52[ax]	1.95[ab]	0.219	not reported
		Chikso	2.21[x]	1.82	1.89[y]	2.23	0.099	
		SEM[2)]	0.141	0.267	0.073	0.137		
Methyl heptanoate	1303	Hanwoo	0.29[abx]	0.39[ax]	0.28[ab]	0.21[bx]	0.028	Fruity, floral, sweet

		Chikso	0.14[by]	0.23[ay]	0.21[a]	0.11[by]	0.015	
		SEM[2)]	0.029	0.025	0.020	0.011		
Methyl hexanoate	1202	Hanwoo	6.70[ax]	6.72[ax]	6.12[ax]	2.65[bx]	0.537	Fruity, ether, sweet
		Chikso	2.11[by]	3.32[ay]	3.74[ay]	1.77[by]	0.212	
		SEM[2)]	0.631	0.319	0.391	0.114		
Methyl octanoate	1406	Hanwoo	0.53[b]	0.92[ax]	0.97[ax]	0.70[abx]	0.059	Green, herbal, sweet
		Chikso	0.46	0.55[y]	0.56[y]	0.51[y]	0.033	
		SEM[2)]	0.047	0.044	0.060	0.035		
Subtotal		Hanwoo	8.84[abx]	10.63[ax]	9.88[ax]	5.67[b]	0.769	
		Chikso	4.92[by]	5.92[aby]	6.40[ay]	4.95[b]	0.281	
		SEM[2)]	0.815	0.579	0.510	0.283		
Fatty acids								
Acetic acid	1496	Hanwoo	0.44[b]	0.51[b]	0.44[b]	3.01[ax]	0.127	Pungent, sour, vinegar
		Chikso	0.63[b]	-	0.74[b]	1.67[ay]	0.175	

		SEM[2)]	0.092	0.061	0.159	0.238		
Butanoic acid	1661	Hanwoo	0.96^{b}	1.71^{bx}	1.77^{b}	3.16^{a}	0.230	Buttery, sweat, fruity
		Chikso	1.09^{c}	0.34^{by}	1.20^{b}	3.13^{a}	0.134	
		SEM[2)]	0.105	0.204	0.239	0.178		
Hexanoic acid	1883	Hanwoo	2.30^{ax}	1.20^{bx}	1.09^{b}	1.58^{ab}	0.181	Fatty, sweat, sour
		Chikso	1.03^{aby}	0.36^{by}	1.04^{ab}	1.39^{a}	0.159	
		SEM[2)]	0.179	0.130	0.161	0.203		
Subtotal		Hanwoo	3.70^{b}	3.42^{bx}	3.31^{b}	7.74^{a}	0.463	
		Chikso	2.74^{b}	0.70^{cy}	2.99^{b}	6.19^{a}	0.385	
		SEM[2)]	0.316	0.389	0.457	0.514		
Furan								
2-Methyloxolane	856	Hanwoo	1.58^{a}	0.31^{b}	0.53^{bx}	0.41^{b}	0.201	not reported
		Chikso	-	0.10	0.09^{y}	-	0.015	
		SEM[2)]	0.252	0.065	0.040	0.111		

Hydrocarbons

1,4-Xylene	1156	Hanwoo	0.24^{y}	0.33	0.23^{y}	0.31	0.035	not reported
		Chikso	0.47^{abx}	0.34^{ab}	0.51^{ax}	0.28^{b}	0.051	
		SEM[2)]	0.056	0.047	0.035	0.031		
2,2,7,7-Tetramethyloctane	983	Hanwoo	-	0.61^{ax}	0.38^{by}	0.17^{c}	0.042	not reported
		Chikso	-	0.16^{by}	1.03^{ax}	0.21^{b}	0.021	
		SEM[2)]	0.000	0.056	0.034	0.011		
2,4-Dimethylhept-1-ene	861	Hanwoo	1.82	3.53	2.94	3.28	0.684	not reported
		Chikso	2.90	2.55	3.30	1.95	0.369	
		SEM[2)]	0.447	0.817	0.361	0.459		
5-Ethyl-2,2,3-trimethylheptane	1034	Hanwoo	0.13^{by}	1.50^{ax}	0.55^{by}	0.47^{b}	0.099	not reported
		Chikso	0.29^{bx}	0.35^{by}	1.33^{ax}	0.43^{b}	0.047	
		SEM[2)]	0.006	0.137	0.065	0.029		
Decane	1002	Hanwoo	1.55	3.16	2.13	2.38	0.409	Alkane
		Chikso	1.73	1.86	2.13	2.29	0.220	

		SEM[2)]	0.129	0.518	0.218	0.314		
Dodecane	1199	Hanwoo	2.39[ax]	0.92[b]	0.70[by]	0.98[bx]	0.089	Alkane
		Chikso	0.90[by]	0.96[b]	1.40[ax]	0.61[cy]	0.059	
		SEM[2)]	0.118	0.060	0.053	0.050		
Toluene	1060	Hanwoo	1.33[by]	1.77[abx]	1.76[ab]	1.96[ax]	0.114	Sweet
		Chikso	2.30[ax]	0.95[by]	2.04[a]	1.17[by]	0.118	
		SEM[2)]	0.156	0.107	0.116	0.068		
Subtotal		Hanwoo	7.46	11.82	8.70[y]	9.54	1.377	
		Chikso	8.59[ab]	7.17[b]	11.74[ax]	6.92[b]	0.769	
		SEM[2)]	0.866	1.719	0.676	0.904		
Ketones								
(3E,5E)-Octa-3,5-dien-2-one	1542	Hanwoo	0.83[a]	0.05[b]	0.08[b]	-	0.030	Fatty, fruity, mushroom
		Chikso	-	-	-	-	0.000	
		SEM[2)]	0.041	0.002	0.006	0.000		

3-Hydroxybutan-2-one	1311	Hanwoo	52.33[by]	80.54[ax]	82.26[ax]	50.55[bx]	1.397	Buttery, fatty, sweet
		Chikso	85.33[ax]	52.54[by]	49.83[by]	13.14[cy]	2.009	
		SEM[2)]	0.627	1.187	0.923	3.053		
4-Methylheptan-2-one	1224	Hanwoo	-	-	-	0.21	0.028	not reported
		Chikso	0.42	0.23	0.51	0.15	0.119	
		SEM[2)]	0.143	0.022	0.074	0.061		
Butane-2,3-dione	994	Hanwoo	5.25[ax]	4.09[bx]	3.97[bx]	4.56[abx]	0.227	Buttery, caramel, pungent
		Chikso	3.93[ay]	2.65[bcy]	3.19[aby]	1.82[cy]	0.231	
		SEM[2)]	0.231	0.250	0.176	0.252		
Propan-2-one	812	Hanwoo	7.25	8.01	8.31	10.90	0.955	Fruity, ethereal, solvent
		Chikso	6.69	7.41	7.41	9.14	0.645	
		SEM[2)]	0.719	0.401	0.526	1.305		
Subtotal		Hanwoo	65.66[by]	92.69[ax]	94.62[ax]	66.21[bx]	1.617	

	Chikso	96.37^{ax}	62.84^{by}	60.93^{by}	24.26^{cy}	2.117
	SEM[2)]	0.606	0.884	1.356	3.348	
Total	Hanwoo	485.68^{ax}	190.70^{cx}	233.65^{bx}	157.73^{dx}	6.417
	Chikso	125.95^{ay}	101.43^{by}	108.90^{by}	78.62^{cy}	1.664
	SEM[2)]	5.728	5.280	4.617	2.427	

[1)] Standard error of the mean (n = 12), [2)] (n = 6).

$^{a-d}$ Different letters within the same row indicate significant differences ($p < 0.05$).

^{x,y} Different letters within the same column indicate significant differences ($p < 0.05$).

LRI, linear retention index; "-", not detected.

The aroma descriptions for each volatile organic compound are from FooDB library (foodb.ca) and literature: (1) Wang et al., 2016; (2) Xu et al., 2021; (3) Stetzer et al., 2008.
