# Supplementary material for: Evaluation of the physicochemical, metabolomic, and sensory characteristics of Chikso and Hanwoo beef during wet aging
Source: Anim Biosci. 2023 Feb 26;36(7):1101–19. doi: 10.5713/ab.23.0001 (PMC10330996; doi:10.5713/ab.23.0001)
Supplement: Supplementary file 4 [file ab-23-0001-Supplementary-Table-2.pdf]

**Table S2.** Volatile flavor compounds (area unit × $10^{6}$) in Hanwoo and Chikso loin during 28 days of aging.

| Compound | LRI | Breed | Aging period (d) | | | | SEM[1)] | Aroma description |
|---|---|---|---|---|---|---|---|---|
| | | | 0 | 7 | 14 | 28 | | |
| ***Alcohols*** | | | | | | | | |
| (3E,5E)-Octa-3,5-dien-2-ol | 1431 | Hanwoo | $0.12^{c}$ | $0.23^{bc}$ | $0.27^{b}$ | $0.46^{a}$ | 0.025 | Green[(1)] |
| | | Chikso | - | - | - | - | 0.000 | |
| | | SEM[2)] | 0.015 | 0.021 | 0.022 | 0.010 | | |
| (E)-Oct-2-en-1-ol | 1626 | Hanwoo | $0.35^{b}$ | $0.44^{b}$ | $0.64^{b}$ | $1.71^{ax}$ | 0.109 | Citrus, green, soapy |
| | | Chikso | 0.23 | 0.35 | 0.40 | $0.20^{y}$ | 0.048 | |
| | | SEM[2)] | 0.062 | 0.046 | 0.068 | 0.134 | | |
| 2-Methylbutan-2-ol | 1020 | Hanwoo | 0.26 | 0.22 | - | 0.07 | 0.055 | Pungent |
| | | Chikso | 0.08 | 0.17 | 0.16 | 0.06 | 0.033 | |
| | | SEM[2)] | 0.070 | 0.044 | 0.037 | 0.011 | | |
| 2-Methylpropan-2-ol | 884 | Hanwoo | 3.26 | 2.21 | 3.50 | 0.58 | 1.720 | Camphor |
| | | Chikso | 0.79 | 1.54 | 1.29 | 0.58 | 0.471 | |

| | | | | | | | | |
|---|---|---|---|---|---|---|---|---|
| | | SEM[2)] | 0.894 | 0.707 | 2.244 | 0.153 | | |
| 3-Methylbutan-1-ol | 1217 | Hanwoo | - | - | $0.20^{b}$ | $0.71^{ax}$ | 0.042 | Fruity, malty, oily |
| | | Chikso | - | - | $0.32^{a}$ | $0.42^{ay}$ | 0.040 | |
| | | SEM[2)] | 0.000 | 0.000 | 0.076 | 0.031 | | |
| Ethanol | 927 | Hanwoo | $1.64^{dx}$ | $4.14^{bx}$ | $2.85^{cy}$ | $23.97^{ax}$ | 0.190 | Alcoholic, ethereal, sweet |
| | | Chikso | $1.03^{dy}$ | $1.94^{cy}$ | $5.83^{bx}$ | $22.65^{ay}$ | 0.090 | |
| | | SEM[2)] | 0.105 | 0.074 | 0.127 | 0.237 | | |
| Heptan-1-ol | 1466 | Hanwoo | $0.29^{b}$ | $0.52^{b}$ | $1.39^{b}$ | $8.69^{ax}$ | 0.668 | Herbal, musty, sweet |
| | | Chikso | 0.57 | 0.72 | 1.41 | $0.98^{y}$ | 0.285 | |
| | | SEM[2)] | 0.113 | 0.115 | 0.434 | 0.916 | | |
| Hexan-1-ol | 1363 | Hanwoo | $1.20^{c}$ | $2.40^{c}$ | $14.41^{b}$ | $90.50^{ax}$ | 2.165 | Ethereal, fruity, sweet |
| | | Chikso | $1.38^{b}$ | $1.75^{b}$ | $12.78^{a}$ | $17.87^{ay}$ | 2.035 | |
| | | SEM[2)] | 0.334 | 0.389 | 3.482 | 2.296 | | |

| | | | | | | | | |
|---|---|---|---|---|---|---|---|---|
| Non-1-en-4-ol | 1671 | Hanwoo | 0.12[b] | 0.34[bx] | 0.83[a] | 1.07[ax] | 0.087 | Grassy, honey[(2)] |
| | | Chikso | 0.10 | 0.08[y] | 0.29 | 0.07[y] | 0.073 | |
| | | SEM[2)] | 0.041 | 0.057 | 0.144 | 0.012 | | |
| Non-2-en-1-ol | 1727 | Hanwoo | - | - | - | 0.88 | 0.077 | Mushroom, melon[(3)] |
| | | Chikso | - | - | - | - | 0.000 | |
| | | SEM[2)] | 0.000 | 0.000 | 0.000 | 0.109 | | |
| Oct-1-en-3-ol | 1460 | Hanwoo | 4.38[b] | 6.51[b] | 8.61[b] | 21.68[ax] | 1.154 | Fishy, fatty, mushroom[(4)] |
| | | Chikso | 3.44 | 5.30 | 6.09 | 2.36[y] | 0.850 | |
| | | SEM[2)] | 0.889 | 0.801 | 0.989 | 1.303 | | |
| Octan-1-ol | 1566 | Hanwoo | 0.19[b] | 0.32[b] | 0.50[b] | 1.68[ax] | 0.133 | Waxy, green, mushroom |
| | | Chikso | 0.35 | 0.51 | 0.59 | 0.41[y] | 0.103 | |
| | | SEM[2)] | 0.064 | 0.071 | 0.120 | 0.182 | | |
| Pent-1-en-3-ol | 1170 | Hanwoo | 0.66[c] | 0.91[bcx] | 1.13[bx] | 1.43[ax] | 0.063 | Buttery, green, fruity |

| | | | | | | | | |
|---|---|---|---|---|---|---|---|---|
| | | Chikso | 0.46[ab] | 0.41[aby] | 0.60[ay] | 0.25[by] | 0.048 | |
| | | SEM[2)] | 0.082 | 0.041 | 0.052 | 0.035 | | |
| Pentan-1-ol | 1260 | Hanwoo | 4.21[c] | 6.34[bx] | 7.81[bx] | 11.29[ax] | 0.400 | Balsamic, oily, sweet |
| | | Chikso | 3.58 | 3.51[y] | 4.05[y] | 1.80[y] | 0.547 | |
| | | SEM[2)] | 0.602 | 0.432 | 0.588 | 0.150 | | |
| Subtotal | | Hanwoo | 16.68[c] | 24.57[bcx] | 42.14[b] | 164.70[ax] | 5.031 | |
| | | Chikso | 12.01[b] | 16.27[by] | 33.82[a] | 47.66[ay] | 3.725 | |
| | | SEM[2)] | 2.895 | 2.066 | 6.377 | 5.006 | | |
| ***Aldehydes*** | | | | | | | | |
| (2E,4E)-Nona-2,4-dienal | 1730 | Hanwoo | 0.13[c] | 0.34[bc] | 0.49[bx] | 0.92[abx] | 0.054 | Fatty, green, nutty |
| | | Chikso | - | 0.23 | 0.23[y] | 0.06[y] | 0.043 | |
| | | SEM[2)] | 0.039 | 0.051 | 0.062 | 0.040 | | |
| (E)-Hept-2-enal | 1349 | Hanwoo | 0.53[cx] | 1.10[abx] | 1.17[ax] | 0.79[bcx] | 0.074 | Fatty, green, almond |
| | | Chikso | 0.11[by] | 0.42[ay] | 0.40[ay] | 0.04[by] | 0.036 | |

| | | | | | | | | |
|---|---|---|---|---|---|---|---|---|
| | | SEM[2)] | 0.094 | 0.061 | 0.027 | 0.015 | | |
| 2-Phenylacetaldehyde | 1676 | Hanwoo | - | - | 0.73$^{b}$ | 2.05$^{a}$ | 0.101 | Cocoa, floral, green |
| | | Chikso | - | - | 1.07$^{b}$ | 2.32$^{a}$ | 0.121 | |
| | | SEM[2)] | 0.000 | 0.000 | 0.167 | 0.147 | | |
| 3-Methylbutanal | 911 | Hanwoo | - | - | 0.99 | 0.82$^{x}$ | 0.068 | Fatty, malty, ethereal |
| | | Chikso | - | - | 0.98$^{a}$ | 0.54$^{by}$ | 0.077 | |
| | | SEM[2)] | 0.000 | 0.000 | 0.134 | 0.058 | | |
| Heptanal | 1203 | Hanwoo | 1.04$^{b}$ | 3.18$^{a}$ | 4.06$^{ax}$ | 4.19$^{ax}$ | 0.392 | Fatty, green, rancid |
| | | Chikso | 0.55$^{b}$ | 2.08$^{a}$ | 2.15$^{ay}$ | 0.49$^{by}$ | 0.324 | |
| | | SEM[2)] | 0.260 | 0.514 | 0.409 | 0.136 | | |
| Hexanal | 1100 | Hanwoo | 9.18$^{cx}$ | 26.79$^{abx}$ | 32.26$^{ax}$ | 21.15$^{bx}$ | 2.079 | Fatty, green, fruity |
| | | Chikso | 1.52$^{by}$ | 15.99$^{ay}$ | 15.75$^{ay}$ | 2.39$^{by}$ | 1.158 | |
| | | SEM[2)] | 0.674 | 2.118 | 1.023 | 2.311 | | |
| Nonanal | 1414 | Hanwoo | 0.45$^{c}$ | 1.06$^{bcy}$ | 1.46$^{b}$ | 2.51$^{ax}$ | 0.145 | Fatty, green, fishy |
| | | Chikso | 0.65$^{b}$ | 2.10$^{ax}$ | 1.75$^{ab}$ | 0.82$^{by}$ | 0.252 | |

| | | | | | | | | |
|---|---|---|---|---|---|---|---|---|
| | | SEM2) | 0.109 | 0.243 | 0.277 | 0.146 | | |
| Octanal | 1308 | Hanwoo | - | 0.24by | 0.32ab | 0.42ax | 0.027 | Fatty, green, citrus |
| | | Chikso | 0.10b | 0.50ax | 0.38a | 0.13by | 0.051 | |
| | | SEM2) | 0.018 | 0.064 | 0.032 | 0.034 | | |
| Pentanal | 996 | Hanwoo | 0.41c | 1.32ax | 1.64ax | 0.80b | 0.071 | Malty, nutty, fruity |
| | | Chikso | - | 0.53by | 0.63ay | - | 0.049 | |
| | | SEM2) | 0.050 | 0.080 | 0.044 | 0.065 | | |
| Subtotal | | Hanwoo | 11.74bx | 34.03ax | 43.12ax | 33.66ax | 2.221 | |
| | | Chikso | 2.94by | 21.85ay | 23.35ay | 6.79by | 1.828 | |
| | | SEM2) | 1.173 | 3.076 | 1.404 | 1.933 | | |
| ***Esters*** | | | | | | | | |
| Ethyl butanoate | 1050 | Hanwoo | - | - | - | 0.15x | 0.004 | Fruity |
| | | Chikso | - | - | - | 0.09y | 0.003 | |
| | | SEM2) | 0.000 | 0.000 | 0.000 | 0.007 | | |
| Methyl 2-hydroxypropanoate | 1337 | Hanwoo | 1.00a | 0.73ab | 0.73abx | 0.64bx | 0.068 | not reported |

| | | | | | | | | |
|---|---|---|---|---|---|---|---|---|
| | | Chikso | $0.78^{a}$ | $0.80^{a}$ | $0.43^{by}$ | $0.56^{by}$ | 0.039 | |
| | | SEM[2)] | 0.079 | 0.061 | 0.047 | 0.014 | | |
| Methyl heptanoate | 1303 | Hanwoo | $0.24^{b}$ | $0.24^{bx}$ | $0.19^{b}$ | $0.45^{ax}$ | 0.043 | Fruity, floral, sweet |
| | | Chikso | $0.33^{a}$ | $0.14^{aby}$ | $0.11^{ab}$ | $0.07^{by}$ | 0.051 | |
| | | SEM[2)] | 0.078 | 0.018 | 0.022 | 0.044 | | |
| Methyl hexanoate | 1202 | Hanwoo | $5.05^{a}$ | $3.73^{abx}$ | $2.68^{bx}$ | $4.22^{ax}$ | 0.335 | Fruity, ether, sweet |
| | | Chikso | $3.80^{a}$ | $2.11^{aby}$ | $1.33^{by}$ | $0.62^{by}$ | 0.434 | |
| | | SEM[2)] | 0.672 | 0.265 | 0.241 | 0.147 | | |
| Methyl octanoate | 1406 | Hanwoo | $0.38^{bx}$ | $0.43^{b}$ | $0.48^{bx}$ | $0.69^{ax}$ | 0.034 | Green, herbal, sweet |
| | | Chikso | $0.28^{y}$ | 0.34 | $0.32^{y}$ | $0.28^{y}$ | 0.031 | |
| | | SEM[2)] | 0.026 | 0.032 | 0.036 | 0.035 | | |
| Subtotal | | Hanwoo | $6.66^{a}$ | $5.12^{abx}$ | $4.07^{bx}$ | $6.15^{ax}$ | 0.348 | |
| | | Chikso | $5.18^{a}$ | $3.40^{aby}$ | $2.21^{by}$ | $1.62^{by}$ | 0.500 | |
| | | SEM[2)] | 0.701 | 0.328 | 0.301 | 0.226 | | |
| ***Fatty acids*** | | | | | | | | |

| | | | | | | | | |
|---|---|---|---|---|---|---|---|---|
| Acetic acid | 1496 | Hanwoo | 0.54c | 1.06bcx. | 2.64b | 5.02a | 0.379 | Pungent, sour, vinegar |
| | | Chikso | 0.35c | 0.33cy | 1.37b | 3.80a | 0.187 | |
| | | SEM2) | 0.079 | 0.093 | 0.340 | 0.477 | | |
| Butanoic acid | 1661 | Hanwoo | 0.66bx | 0.54bx | 1.44bx | 2.97ax | 0.216 | Buttery, sweat, fruity |
| | | Chikso | 0.19cy | 0.16cy | 0.74by | 1.70ay | 0.087 | |
| | | SEM2) | 0.104 | 0.019 | 0.131 | 0.283 | | |
| Hexanoic acid | 1883 | Hanwoo | 2.05b | 1.48bx | 2.82b | 6.41ax | 0.700 | Fatty, sweat, sour |
| | | Chikso | 1.34ab | 0.30by | 1.32ab | 1.83ay | 0.244 | |
| | | SEM2) | 0.277 | 0.013 | 0.392 | 0.933 | | |
| Subtotal | | Hanwoo | 3.24b | 3.07bx | 6.90bx | 14.40ax | 1.280 | |
| | | Chikso | 1.88bc | 0.80cy | 3.43by | 7.33ay | 0.485 | |
| | | SEM2) | 0.418 | 0.114 | 0.847 | 1.686 | | |
| ***Furan*** | | | | | | | | |

| | | | | | | | | |
|---|---|---|---|---|---|---|---|---|
| 2-Methyloxolane | 856 | Hanwoo | 0.13[bx] | 0.29[a] | 0.22[ab] | 0.26[ax] | 0.023 | not reported |
| | | Chikso | 0.05[y] | 0.41 | 0.29 | 0.03[y] | 0.088 | |
| | | SEM[2)] | 0.012 | 0.124 | 0.029 | 0.013 | | |
| ***Hydrocarbons*** | | | | | | | | |
| 1,4-Xylene | 1156 | Hanwoo | 0.07 | 0.05 | 0.05[y] | 0.05[y] | 0.011 | not reported |
| | | Chikso | 0.09 | 0.10 | 0.09[x] | 0.08[x] | 0.008 | |
| | | SEM[2)] | 0.011 | 0.013 | 0.005 | 0.005 | | |
| 2,4-Dimethylhept-1-ene | 861 | Hanwoo | 0.26 | 0.28[y] | 0.27 | 0.24[y] | 0.072 | not reported |
| | | Chikso | 0.35 | 0.71[x] | 0.59 | 0.67[x] | 0.114 | |
| | | SEM[2)] | 0.064 | 0.094 | 0.109 | 0.107 | | |
| Decane | 1002 | Hanwoo | 0.36 | 0.36 | 0.38 | 0.44 | 0.042 | Alkane |
| | | Chikso | 0.21 | 0.48 | 0.44 | 0.46 | 0.091 | |
| | | SEM[2)] | 0.057 | 0.070 | 0.087 | 0.065 | | |
| Dodecane | 1199 | Hanwoo | 0.26[b] | 0.28[by] | 0.29[b] | 0.71[ax] | 0.027 | Alkane |
| | | Chikso | 0.22 | 0.37[x] | 0.39 | 0.33[y] | 0.049 | |

| | | | | | | | | |
|---|---|---|---|---|---|---|---|---|
| | | SEM[2)] | 0.046 | 0.022 | 0.056 | 0.024 | | |
| Toluene | 1060 | Hanwoo | 0.55[ax] | 0.55[ax] | 0.52[ax] | 0.41[b] | 0.015 | Sweet |
| | | Chikso | 0.36[by] | 0.33[by] | 0.46[ay] | 0.37[b] | 0.012 | |
| | | SEM[2)] | 0.016 | 0.016 | 0.011 | 0.012 | | |
| Subtotal | | Hanwoo | 1.50 | 1.76 | 1.69 | 1.96 | 0.136 | |
| | | Chikso | 1.23[b] | 2.00[ab] | 2.46[a] | 2.13[ab] | 0.243 | |
| | | SEM[2)] | 0.152 | 0.190 | 0.246 | 0.187 | | |
| ***Ketones*** | | | | | | | | |
| (3E,5E)-Octa-3,5-dien-2-one | 1542 | Hanwoo | 0.06[c] | 0.12[bcx] | 0.15[bx] | 0.25[a] | 0.019 | Fatty, fruity, mushroom |
| | | Chikso | 0.04 | 0.05[y] | 0.06[y] | - | 0.013 | |
| | | SEM[2)] | 0.010 | 0.016 | 0.020 | 0.016 | | |
| 3-Hydroxybutan-2-one | 1311 | Hanwoo | 17.71[cx] | 20.31[cx] | 63.78[a] | 41.90[b] | 4.335 | Buttery, fatty, sweet |
| | | Chikso | 7.70[cy] | 12.96[bcy] | 59.77[a] | 35.3[ab] | 5.431 | |
| | | SEM[2)] | 0.465 | 0.908 | 9.179 | 3.360 | | |

| | | | | | | | | |
|---|---|---|---|---|---|---|---|---|
| Butane-2,3-dione | 994 | Hanwoo | $0.98^{cx}$ | $1.22^{bc}$ | $3.44^{a}$ | $1.92^{b}$ | 0.186 | Buttery, caramel, pungent |
| | | Chikso | $0.59^{by}$ | $1.02^{b}$ | $3.68^{a}$ | $1.49^{b}$ | 0.251 | |
| | | SEM[2)] | 0.082 | 0.056 | 0.401 | 0.154 | | |
| Propan-2-one | 812 | Hanwoo | 2.99 | 3.03 | $2.35^{y}$ | 3.56 | 0.306 | Fruity, ethereal, solvent |
| | | Chikso | 3.13 | 4.33 | $3.59^{x}$ | 3.74 | 0.521 | |
| | | SEM[2)] | 0.379 | 0.672 | 0.289 | 0.229 | | |
| Subtotal | | Hanwoo | $21.74^{cx}$ | $24.68^{cx}$ | $69.73^{a}$ | $47.63^{b}$ | 4.579 | |
| | | Chikso | $11.46^{cy}$ | $18.36^{bcy}$ | $67.10^{a}$ | $40.55^{b}$ | 5.666 | |
| | | SEM[2)] | 0.399 | 1.453 | 9.648 | 3.284 | | |
| Total | | Hanwoo | $62.22^{cx}$ | $94.09^{bx}$ | $168.54^{b}$ | $269.07^{ax}$ | 8.899 | |
| | | Chikso | $35.38^{cy}$ | $63.88^{bcy}$ | $133.31^{a}$ | $106.96^{aby}$ | 11.097 | |
| | | SEM[2)] | 4.874 | 6.087 | 17.955 | 4.635 | | |

[1)] Standard error of the mean (n = 12), [2)] (n = 6).

[a-d] Different letters within the same row indicate significant differences ($p < 0.05$).

[x,y] Different letters within the same column indicate significant differences ($p < 0.05$).

LRI, linear retention index; -, not detected.

The aroma descriptions for each volatile organic compound are from FooDB library (foodb.ca) and literature: (1) Wang et al., 2016; (2) Xu et al., 2021; (3) Sekhon et al., 2010; (4) Stetzer et al., 2008.
