# Supplementary material for: Evaluation of the physicochemical, metabolomic, and sensory characteristics of Chikso and Hanwoo beef during wet aging
Source: Anim Biosci. 2023 Feb 26;36(7):1101–19. doi: 10.5713/ab.23.0001 (PMC10330996; doi:10.5713/ab.23.0001)
Supplement: Supplementary file 5 [file ab-23-0001-Supplementary-Table-3.pdf]

Table S3. Pearson correlation coefficient (n = 30) between meat quality traits and metabolites in Hanwoo and Chikso beef cuts.

Item	pH	CIE L^*	CIE a^*	CIE b^*	SF	MFI
Rump cut						
Acetate	-0.40	-0.01	-0.41	-0.45	-0.17	**0.60**
Alanine	-0.46	-0.12	**-0.57**	**-0.62**	-0.31	**0.73**
Anserine	-0.28	0.45	-0.31	-0.14	**-0.60**	0.27
Asparagine	-0.48	-0.02	**-0.71**	**-0.64**	-0.36	**0.80**
Carnosine	-0.13	0.40	-0.44	-0.16	**-0.57**	0.23
Creatine	0.07	-0.23	-0.03	-0.21	0.01	0.23
Ethanol	-0.35	-0.44	-0.42	**-0.53**	0.27	0.38
Formate	**-0.55**	0.17	-0.33	-0.28	0.03	0.32
Fumarate	**-0.53**	-0.08	-0.38	-0.47	-0.23	**0.54**
Glutamate	-0.46	-0.07	**-0.74**	**-0.67**	-0.39	**0.78**
Glycine	-0.08	-0.07	-0.31	-0.33	-0.21	0.17
Hypoxanthine	-0.50	0.32	-0.49	-0.42	-0.50	**0.62**
IMP	**0.64**	-0.30	**0.62**	0.48	**0.55**	**-0.65**
Inosine	0.14	-0.42	-0.25	-0.41	-0.24	0.28
Isoleucine	-0.48	-0.07	**-0.74**	**-0.66**	-0.40	**0.76**
Lactate	-0.14	-0.14	-0.17	-0.32	-0.12	0.38
L-Carnitine	0.00	-0.33	-0.16	-0.37	0.11	0.42
Leucine	-0.47	-0.08	**-0.77**	**-0.70**	-0.41	**0.81**
Methionine	-0.45	-0.10	**-0.76**	**-0.71**	-0.42	**0.82**
N,N-Dimethylglycine	-0.21	-0.01	-0.30	-0.32	-0.12	0.46

Niacinamide	-0.23	-0.16	**-0.59**	**-0.61**	**-0.51**	**0.65**
Phenylalanine	**-0.54**	0.00	**-0.80**	**-0.69**	-0.43	**0.83**
Taurine	0.01	0.00	0.06	0.00	0.05	0.02
Tyrosine	-0.29	-0.17	**-0.53**	**-0.54**	-0.34	**0.59**
Uridine	**-0.55**	0.17	**-0.67**	**-0.53**	**-0.53**	**0.80**
Valine	**-0.52**	-0.05	**-0.77**	**-0.70**	-0.44	**0.86**
o-Acetylcarnitine	0.17	-0.09	0.39	0.36	0.45	**-0.66**
Loin cut						
Acetate	**-0.78**	0.08	-0.41	-0.29	-0.28	0.46
Alanine	**-0.68**	-0.03	**-0.62**	-0.49	-0.23	0.45
Anserine	0.22	0.02	0.13	0.02	0.33	-0.17
Asparagine	**-0.82**	-0.10	**-0.63**	**-0.51**	-0.31	**0.63**
Carnosine	0.06	-0.06	-0.37	-0.18	0.35	-0.01
Creatine	0.27	0.15	-0.19	0.00	0.23	-0.30
Ethanol	-0.39	-0.02	-0.23	-0.19	0.24	0.39
Formate	**-0.73**	0.05	-0.38	-0.28	-0.24	0.40
Fumarate	**-0.76**	-0.03	-0.41	-0.34	-0.23	0.31
Glutamate	**-0.81**	0.01	**-0.55**	-0.41	-0.44	**0.60**
Glycine	0.10	0.24	0.21	0.17	-0.12	-0.18
Hypoxanthine	**-0.54**	0.16	**-0.50**	-0.39	-0.39	0.20
IMP	0.47	-0.07	0.19	0.30	**0.69**	-0.26
Inosine	**0.57**	-0.07	0.22	0.27	0.41	-0.35
Isoleucine	**-0.79**	-0.19	**-0.67**	**-0.58**	-0.37	**0.56**
Lactate	-0.27	0.11	-0.47	-0.22	0.08	-0.04

L-Carnitine	-0.17	-0.23	**-0.54**	-0.41	0.08	**0.59**
Leucine	**-0.79**	-0.15	**-0.71**	**-0.59**	-0.40	**0.61**
Methionine	**-0.64**	-0.09	**-0.60**	**-0.53**	-0.20	**0.56**
N,N-Dimethylglycine	-0.47	0.17	-0.48	-0.24	-0.11	0.31
Niacinamide	**0.58**	-0.17	-0.03	-0.05	0.31	-0.20
Phenylalanine	**-0.79**	-0.19	**-0.72**	**-0.59**	-0.39	**0.62**
Taurine	0.06	-0.03	-0.02	0.07	-0.17	-0.16
Tyrosine	-0.49	-0.45	**-0.52**	-0.46	-0.29	**0.73**
Uridine	-0.36	0.13	-0.04	-0.09	-0.32	0.13
Valine	**-0.83**	-0.10	**-0.67**	**-0.51**	-0.38	**0.65**
o-Acetylcarnitine	0.39	-0.06	0.05	0.10	0.30	-0.37

Pearson correlation coefficients with bold letters indicate that the coefficients showed $|r| > 0.05$ and $p < 0.01$.

MFI, myofibrillar fragmentation index; SF, shear force.
