# Supplementary material for: Evaluation of the physicochemical, metabolomic, and sensory characteristics of Chikso and Hanwoo beef during wet aging
Source: Anim Biosci. 2023 Feb 26;36(7):1101–19. doi: 10.5713/ab.23.0001 (PMC10330996; doi:10.5713/ab.23.0001)
Supplement: Supplementary file 6 [file ab-23-0001-Supplementary-Table-4.pdf]

Table S4. Pearson correlation coefficient (n = 24) between metabolites of Hanwoo and Chikso beef cuts and their sensor values of electronic tongue.

Item	AHS	PKS	CTS	NMS	CPS	ANS	SCS
Rump cut							
Acetate	0.35	0.35	0.20	0.31	-0.26	-0.06	0.32
Alanine	0.16	0.25	0.07	0.34	-0.32	-0.14	0.14
Anserine	-0.11	0.01	0.02	0.28	-0.48	-0.48	-0.15
Asparagine	0.20	0.24	0.22	0.26	-0.27	-0.10	0.15
Carnosine	-0.11	-0.04	-0.01	0.09	-0.28	-0.40	-0.11
Creatine	-0.07	0.14	0.04	-0.03	-0.22	0.01	-0.09
Ethanol	-0.22	-0.23	0.12	-0.26	-0.04	0.08	-0.27
Formate	0.25	0.07	0.13	0.32	-0.18	-0.22	0.22
Fumarate	0.05	0.09	0.09	0.27	-0.25	-0.17	0.02
Glutamate	0.09	0.18	0.15	0.23	-0.33	-0.16	0.04
Glycine	-0.42	-0.23	0.16	-0.09	-0.26	-0.32	-0.49
Hypoxanthine	0.30	0.34	0.02	**0.55**	-0.29	-0.28	0.31
IMP	-0.20	-0.17	0.01	**-0.54**	0.36	0.41	-0.20
Inosine	-0.01	0.29	0.17	0.03	0.08	0.31	-0.06
Isoleucine	0.05	0.19	0.13	0.22	-0.34	-0.17	0.00
Lactate	0.04	0.22	0.10	0.20	-0.27	-0.05	0.01
L-Carnitine	0.35	0.50	0.20	0.16	0.07	0.41	0.33
Leucine	0.12	0.22	0.18	0.24	-0.31	-0.11	0.07
Methionine	0.12	0.23	0.14	0.27	-0.30	-0.12	0.08
N,N-Dimethylglycine	0.07	0.14	0.25	0.07	-0.11	-0.05	0.03

Niacinamide	0.16	0.36	0.21	0.28	-0.13	0.08	0.11
Phenylalanine	0.12	0.19	0.18	0.28	-0.32	-0.17	0.06
Taurine	0.06	-0.06	-0.03	0.10	-0.32	-0.22	0.05
Tyrosine	-0.06	0.16	0.03	0.13	-0.28	-0.09	-0.08
Uridine	0.08	0.19	0.07	0.33	-0.44	-0.33	0.05
Valine	0.19	0.26	0.18	0.32	-0.28	-0.11	0.14
o-Acetylcarnitine	**-0.53**	**-0.62**	-0.17	-0.32	0.24	-0.05	**-0.52**
Loin cut							
Acetate	**-0.60**	-0.29	0.16	-0.10	-0.11	-0.22	**-0.60**
Alanine	**-0.53**	-0.27	0.10	0.11	-0.14	-0.23	**-0.54**
Anserine	0.30	0.11	-0.23	0.12	0.05	0.02	0.30
Asparagine	**-0.61**	-0.35	0.28	-0.03	-0.20	-0.23	**-0.62**
Carnosine	0.11	-0.32	-0.02	0.07	-0.30	-0.21	0.10
Creatine	0.26	-0.13	-0.14	0.08	-0.18	-0.15	0.26
Ethanol	-0.49	-0.49	0.18	-0.02	-0.35	-0.44	-0.50
Formate	**-0.59**	-0.30	0.10	-0.11	-0.12	-0.31	**-0.60**
Fumarate	**-0.69**	-0.44	0.19	-0.02	-0.31	-0.44	**-0.70**
Glutamate	**-0.58**	-0.23	0.19	-0.09	-0.06	-0.19	**-0.59**
Glycine	0.28	0.34	-0.18	-0.02	0.38	0.24	0.28
Hypoxanthine	-0.30	-0.14	0.11	0.04	-0.08	-0.21	-0.31
IMP	0.18	-0.35	-0.03	0.03	-0.43	-0.23	0.17
Inosine	0.41	-0.06	-0.06	0.07	-0.20	-0.05	0.40
Isoleucine	**-0.54**	-0.29	0.27	0.01	-0.15	-0.17	**-0.55**
Lactate	-0.18	-0.39	0.00	0.07	-0.39	-0.37	-0.19

L-Carnitine	-0.12	0.05	0.07	0.30	0.16	0.25	-0.12
Leucine	**-0.51**	-0.24	0.30	0.01	-0.12	-0.11	**-0.52**
Methionine	-0.47	-0.27	0.24	0.02	-0.13	-0.21	-0.48
N,N-Dimethylglycine	-0.34	-0.41	0.17	-0.06	-0.29	-0.34	-0.35
Niacinamide	**0.51**	0.14	-0.03	0.29	-0.01	0.16	**0.51**
Phenylalanine	**-0.54**	-0.27	0.31	0.02	-0.15	-0.13	**-0.54**
Taurine	0.08	-0.03	-0.03	0.11	-0.10	-0.07	0.08
Tyrosine	-0.47	-0.22	0.26	0.18	-0.22	-0.11	-0.48
Uridine	-0.19	-0.20	0.27	-0.11	-0.13	-0.20	-0.20
Valine	**-0.60**	-0.30	0.28	-0.02	-0.17	-0.18	**-0.61**
o-Acetylcarnitine	0.33	-0.07	-0.12	0.15	-0.27	-0.18	0.33

Pearson correlation coefficients with bold letters indicate that the coefficients showed $|r| > 0.05$ and $p < 0.01$.

The electronic tongue sensors AHS, CTS, NMS, ANS, and SCS respond to sour, salty, umami, sweet, and bitterness, respectively, whereas PKS and CPS represent universal taste intensity.
